# Smart Organic–Inorganic Copolymer Nanoparticles Distinguish Between Microglia and Cancer Cells for Synergistic Immunotherapy in Glioma

**DOI:** 10.1002/advs.202500882

**Published:** 2025-04-29

**Authors:** Shiming Zhang, Kun Shang, Lidong Gong, Qian Xie, Jianfei Sun, Meng Xu, Xunbin Wei, Zhaoheng Xie, Xinyu Liu, Hao Tang, Zhengren Xu, Wei Wang, Haihua Xiao, Zhiqiang Lin, Hongbin Han

**Affiliations:** ^1^ Institute of Medical Technology Peking University Health Science Center Beijing 100190 P. R. China; ^2^ Department of Nuclear Medicine Peking University People's Hospital Beijing 100190 P. R. China; ^3^ Institute of Systems Biomedicine Department of Pathology Department of Biophysics School of Basic Medical Sciences Peking University Health Science Center Beijing 100191 P. R. China; ^4^ Division of Nephrology Peking University Third Hospital Beijing 100191 P. R. China; ^5^ Jiangsu Key Laboratory for Biomaterials and Devices School of Biological Science and Medical Engineering Southeast University Nanjing 210096 P. R. China; ^6^ Department of Computer Science Peking University Beijing 100191 P. R. China; ^7^ State Key Laboratory of Natural and Biomimetic Drugs School of Pharmaceutical Sciences Peking University Beijing 100191 P. R. China; ^8^ Department of Rehabilitation Radiology Beijing Rehabilitation Hospital Capital Medical University Beijing 100144 P. R. China; ^9^ Beijing National Laboratory for Molecular Science State Key Laboratory of Polymer Physics and Chemistry Institute of Chemistry Chinese Academy of Science Beijing 100190 P. R. China; ^10^ Department of Radiology Peking University Third Hospital Institute of Medical Technology Peking University Health Science Center Beijing 100190 P. R. China

**Keywords:** brain extracellular space, glioma, immunotherapy, membranolytic activity, STING pathway

## Abstract

The stimulator of interferon genes (STING) pathway has emerged as a new immunotherapy strategy with potent local stimulation specificity, showing promising potential to counteract the immunosuppression in glioma. Herein, a tumor microenvironment (TME) responsive nanoagonists are developed based on an organic–inorganic copolymer composed of the polymer PC6AB coupled with manganous phosphate ionic oligomers (MnP). The degradation of nanoagonists into PC6AB and MnP in the acidic TME enables spatiotemporal control of their delivery to tumor cells and immune cells, respectively. PC6AB with membranolytic activity selectively interacts with tumor cell membranes to induce immunogenic cell death, while manganese metal can activate the STING pathway in immune cells and trigger downstream immunostimulatory signals. Nanoagonists can stimulate robust antitumor immunity after local injection into the brain extracellular space (ECS), showing significant therapeutic efficacy in mouse glioma. Nanoagonists can achieve spatiotemporal orchestration of STING activation in response to TME and enhance immune response against “cold” solid tumors, providing a promising approach for clinical immunotherapy.

## Graphical abstract

The tumor microenvironment‐responsive nanoagonists are developed using an organic–inorganic copolymer composed of the polymer (PC6AB) and manganous phosphate ionic oligomers (MnP). PC6AB with membranolytic activity selectively interacts with tumor cell membranes to induce immunogenic cell death, while MnP activates the STING pathway in immune cells, triggering downstream immunostimulatory signals.

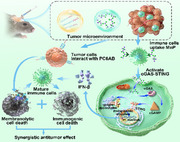

## 1 Introduction

Immunotherapy has revolutionized cancer treatment, gathering significant attention for its ability to induce antigen‐specific immunological memory and control tumor growth, particularly through immune checkpoint inhibitors (ICIs).^[^ ^1^, ^2^, ^3^ ^]^ However, its application in solid tumors, including glioma, remains challenging due to the highly immunosuppressive tumor microenvironment (TME) in the brain, which evades T cell‐mediated immune responses.^[^ ^4^, ^5^ ^]^ The immunosuppressive “cold” TME in glioblastoma multiforme often results in poor efficacy and low response rates with substantial inter‐patient variability.^[^ ^6^ ^]^ Therefore, restoring and stimulating immune cell function has become a promising strategy to overcome intratumoral immunosuppression and foster antitumor immunity.^[^ ^7^ ^]^

Recent studies highlight the critical role of the cyclic guanosine monophosphate‐adenosine monophosphate synthase‐stimulator of interferon genes (cGAS‐STING) pathway in reprogramming the immunosuppressive TME and enhancing ICIs therapies.^[^ ^8^, ^9^ ^]^ The STING pathway is an important component of innate immunity and has severed as a new immunotherapy target for potent local immune stimulation.^[^ ^10^ ^]^ Briefly, cGAS induces STING oligomerization and activates TANK‐binding kinase 1 (TBK1) and interferon regulatory factor 3 (IRF3), triggering various immunostimulatory signals in immune cells.^[^ ^11^, ^12^ ^]^ However, recent studies suggest that the clinical translation of STING agonists has been limited by issues such as unregulated downstream pathway, poor cellular permeability, inadequate stability, suboptimal pharmacokinetics, and off‐target effects.^[^ ^13^, ^14^, ^15^, ^16^, ^17^ ^]^ To address these limitations, immunotherapy has been combined with radiation therapy, immune adjuvant,^[^ ^18^ ^]^ photodynamic therapy,^[^ ^19^ ^]^ and chemotherapy to enhance antitumor efficacy.^[^ ^20^, ^21^ ^]^ A strategy combining radiosensitization with STING pathway activation has been proposed by fabricating a novel lanthanide‐doped radiosensitizer‐based metal‐phenolic network, significantly benefiting dendritic cell maturation and antitumor therapeutics in primary tumors.^[^ ^22^ ^]^ Chemotherapeutic platinum complexes and a STING agonist, a cyclic seven‐membered ring (PC7A), encapsulated into pH‐responsive nanoparticles for multimodal therapeutically enhanced chemotherapy and immunotherapy can almost completely eradicate colorectal tumors inside the mouse model.^[^ ^23^ ^]^ Nevertheless, the cellular microenvironment of glioma is a physiologically complex niche with diverse cell populations, including tumor cells and glial cells that exert immune function.^[^ ^24^, ^25^, ^26^, ^27^ ^]^ In combination therapy, it is important for STING agonists to be specifically delivered to glial cells and for cytotoxic stimuli to act specifically on tumor cells, which can enhance immune activation and reduce non‐specific toxicity.^[^ ^28^ ^]^ Therefore, it is necessary to explore appropriate STING agonists and design combination therapy strategies to amplify antitumor immune response and improve treatment efficacy.

A block copolymer (PC6AB) with membranolytic activity has been reported to self‐assemble into neutral‐charged nanoparticles under physiological pH. These nanoparticles exhibit low toxicity in healthy tissues and demonstrate potent membranolytic activity after transitioning sharply to a protonated state under the acidic conditions of tumors.^[^ ^29^ ^]^ Previous studies have primarily concentrated on the selective toxicity of nanoparticles to acidic tumor tissues while minimizing toxicity to normal tissues. However, there is a notable lack of research focused on the differential cytotoxicity of nanoparticles in the acidic TME, particularly highlighting their ability to selectively target tumor cells while sparing immune and other normal cells. Recent studies have shown that manganese is a potent activator of the cGAS‐STING, which can synergize with STING agonists, such as cyclic dinucleotide (CDN).^[^ ^30^ ^]^ This combination significantly enhances STING activation and helps reverse immune suppression within the TME. Manganese, an inorganic metal, is typically modified with proteins or organic molecules to mitigate potential adverse effects.^[^ ^31^, ^32^ ^]^ However, simply encapsulating manganese within polymers, without chemical coupling, does not guarantee the desired immune responses due to the risk of non‐specific leakage during transport.^[^ ^33^ ^]^ In recent years, it has been reported that ion oligomers can connect with the amino groups of polymers to form a continuous hybrid network of organic–inorganic copolymers, which are mainly applied in the field of materials science to provide environmentally friendly and biodegradable alternatives to traditional plastics.^[^ ^34^, ^35^, ^36^ ^]^ However, research data on organic–inorganic copolymers in the field of life sciences is still scarce. Further studies are required to demonstrate that organic–inorganic copolymers can enable advanced biomedical systems to achieve enhanced therapeutic efficacy, biocompatibility, and multifunctionality. Developing effective nanoagonists that achieve spatiotemporal control of immune stimulation in combination with chemotherapy remains a challenge. This capability is urgently needed to induce robust, tumor‐specific responses but is still underexplored.

**Scheme 1:**
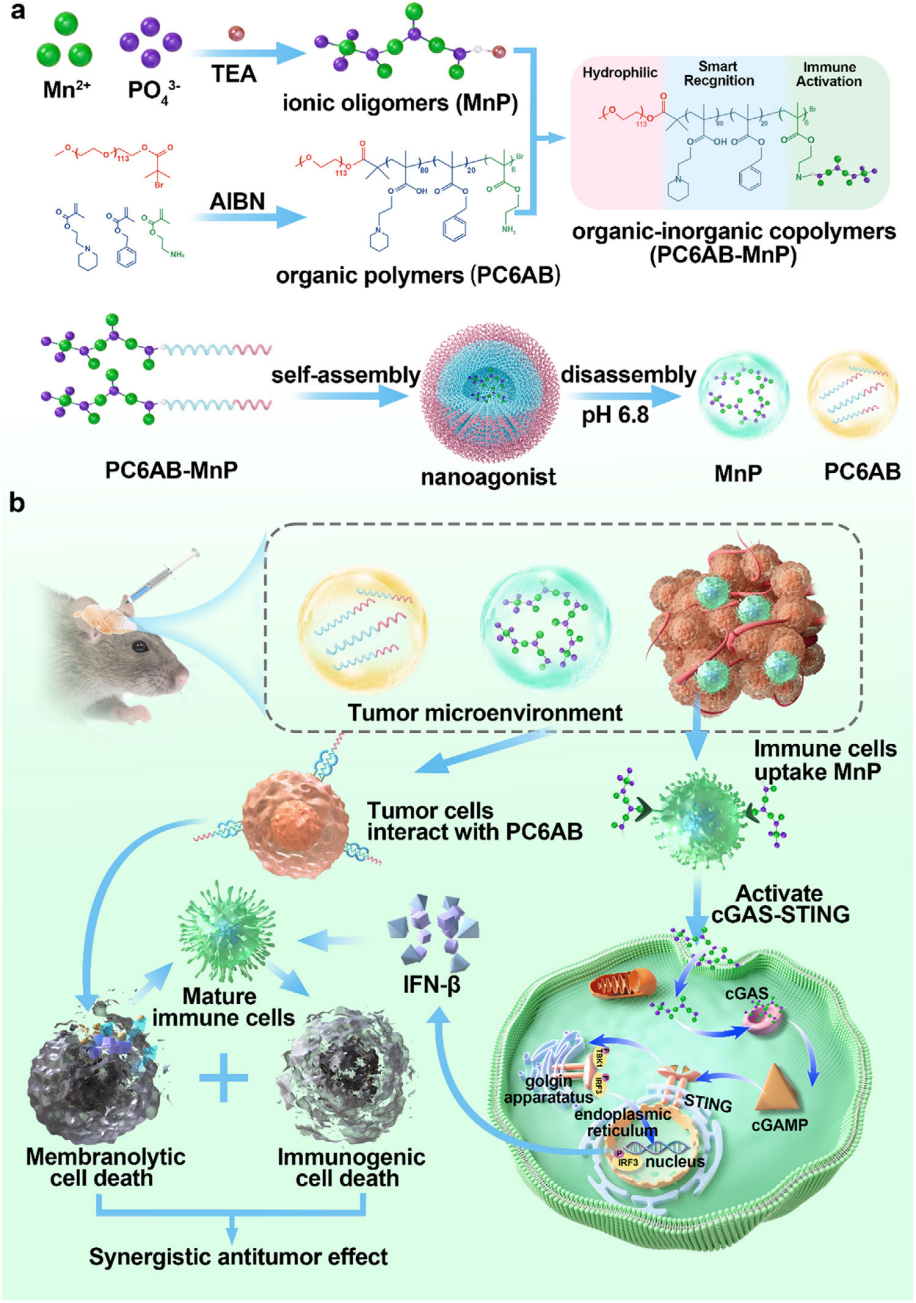
On‐demand activation nanoagonists for cancer immunotherapy. a) Nanoagonists were self‐assembled from polymer PC6AB conjugated with MnP. Nanoagonists are composed of 3 functional components: (1) PEG forms a hydrophilic outer shell, (2) C6A and Bn form a hydrophobic core with membranolytic selectivity, (3) MnP has an immune‐activating function. b) The administered nanoagonists rapidly disassembled into PC6AB and MnP in the acidic TME. PC6AB exhibited a strong affinity for the tumor cell membranes, triggering potent membranolytic activity that leads to tumor cell destruction. Meanwhile, the released MnP was taken up by immune cells, activating the cGAS‐STING pathway to enhance immunotherapeutic efficacy. The combination of immunotherapy and chemotherapy synergistically produces potent antitumor effect.

Herein, we have developed a TME‐activatable nanoagonist based on membranolytic block copolymer (PC6AB) coupled with ionic oligomers (MnP). This nanoagonist, designed to respond to the TME, utilized PC6AB's membranolytic activity to induce tumor cell death, thereby providing a chemotherapy effect, while MnP activated immune cells and triggered the STING pathway, generating an immunotherapy effect. By spatiotemporally coordinating the specific lysis of tumor cell membranes and facilitating the delivery of MnP to immune cells, the nanoagonists enhanced both chemotherapeutic efficacy and immunotherapy, resulting in a synergistic antitumor effect. The nanoagonist consists of three independently controlled functional components. The first is the immune activation component, composed of MnP encapsulated in a hydrophobic core, which simultaneously activates the cGAS‐STING pathway, modulates the TME, and reverses tumor immunosuppression. The second is an intelligent recognition component composed of hexamethylenimine (C6A) and benzyl (Bn) blocks, which exhibits membranolytic selectivity by recognizing acidic phospholipids on tumor cell membranes without affecting immune cells. The third is a hydrophilic component composed of polyethylene glycol (PEG) as a hydrophilic shell to maintain the colloidal stability of the nanoagonists, improving drug bioavailability and therapeutic efficacy. The antitumor immune efficacy of the nanoagonists was investigated in a mouse glioma model after local administration to the brain extracellular space (ECS) via convection‐enhanced delivery (CED). Due to the inclusion of manganese as a magnetic resonance imaging (MRI) contrast agent and labeling with Cyanine5 (Cy5) as a near‐infrared imaging (NIRI) agent, the nanoagonist enables imaging‐guided synergistic therapy through dual‐mode fluorescent and magnetic imaging, allowing real‐time monitoring of drug delivery. Together, this study demonstrated that nanoagonists activated the cGAS‐STING pathway, synergized immunotherapy and chemotherapy to alleviate glioma, and improved antitumor immune efficacy antitumor immunity, providing emerging opportunities for enhancing the efficacy of immunotherapies (**Scheme** **1**).

## 2 Results and Discussion

### 2.1 Construction and Characterization of the Nanoagonists

**Figure 1:**
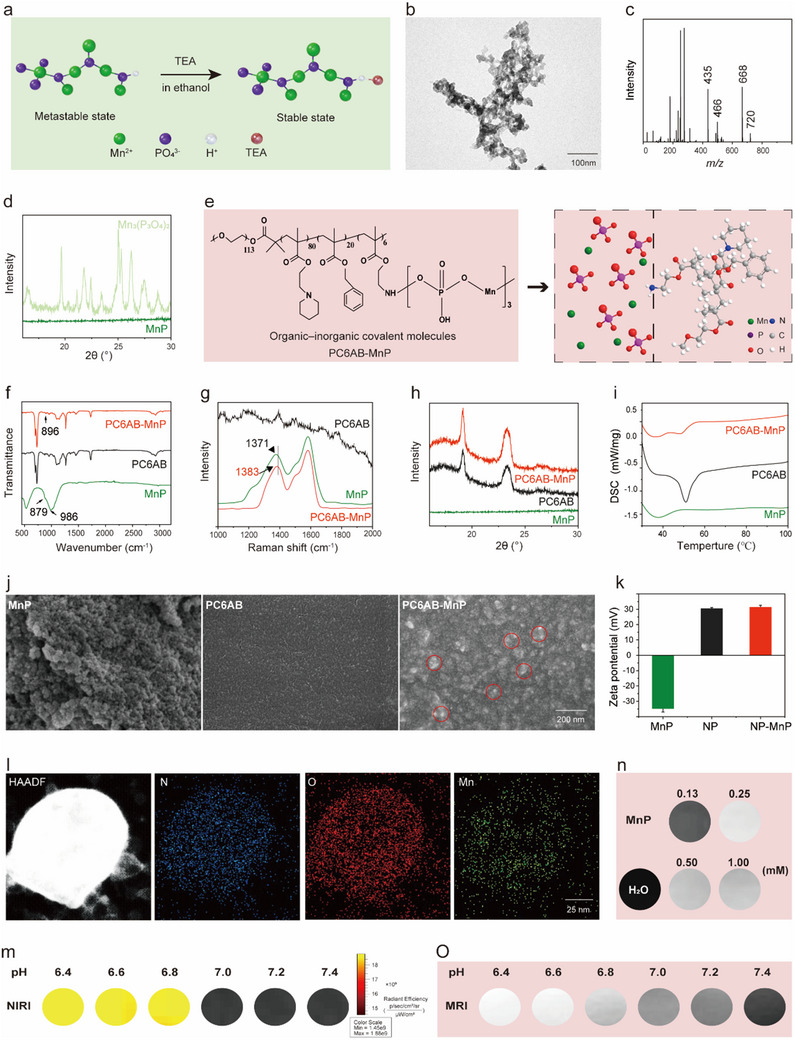
Characterization of the NP‐MnP. a) Schematic diagram of the capping strategy and reaction conditions for producing MnP oligomers. b) TEM image of the MnP for illustrating the morphological details. c) Mass spectrum of MnP. d) XRD patterns of MnP and Mn_3_(PO_4_)_2_. e) Schematic illustrations of the molecular structures of PC6AB‐MnP hybrid molecule. f) ATR‐FTIR spectra of MnP, PC6AB, and PC6AB‐MnP. g) Raman spectra of MnP, PC6AB, and PC6AB‐MnP. h) XRD patterns of MnP, PC6AB, and PC6AB‐MnP. i) DSC patterns of MnP, PC6AB, and PC6AB‐MnP. j) SEM images of the MnP, PC6AB, and PC6AB‐MnP. The red circles represent aggregated MnP. k) Zeta potential of MnP, NP, and MnP‐NP. All data were expressed as mean ± SD (n = 3). l) Elemental mapping of NP‐MnP. m) NIRI of NP‐MnP at pH 6.4, 6.8, 7.0, 7.2, and 7.4. n) T1‐weighted MRI of MnP at various concentrations. o) T1‐weighted MRI of NP‐MnP at pH 6.4, 6.8, 7.0, 7.2, and 7.4.

MnP was prepared by dissolving manganese ions and phosphoric acid in ethanol using trimethylamine (TEA) as the end‐capping agent.^[^ ^36^ ^]^ Ethanol, as a low dielectric constant solvent, promotes the formation of hydrogen bonds between the nitrogen of TEA and the protonated phosphates, leading to the formation of ionic oligomers rather than classical manganese phosphate crystallization (**Figure** **1**a). The MnP contained cross‐linked oligomers with branched structures, as shown by transmission electron microscopy (TEM) (Figure 1b; Figure S1, Supporting Information). Electrospray ionization mass spectrometry (ESI‐MS) of MnP displayed complexity due to the polydispersity of MnP with varying degrees of polymerization. Peaks at 435, 499, 668, and 720 *m/z*, which exceeded the molecular weight of the MnP monomer, indicated that MnP existed in a polymerized state (Figure 1c). X‐ray photoelectron spectroscopy (XPS) spectrum analysis showed corresponding N1s peaks on MnP revealed the presence of C─N and N─H bonds in MnP, indicating the effective incorporation of TEA into the MnP (Figure S2, Supporting Information). The powder X‐ray diffraction (XRD) patterns indicated that MnP was in an amorphous state, in contrast to the crystalline state of manganese phosphate (Figure 1d). These findings demonstrated that MnP is not a manganese phosphate, but rather an inorganic ionic polymer terminated with TEA and potential capping effects. Attenuated total reflectance Fourier transform infrared spectroscopy (ATR‐FTIR) revealed that the characteristic peak of C─N stretching vibration in MnP was at 986 cm^−1^, indicating the presence and potential capping effect of TEA (Figure S3, Supporting Information). The broadness of the C‐N peak may be attributed to the hydrogen bonds between the nitrogen in TEA and the oxygen in MnP, which also provided further evidence for the synthesis of MnP.^[^ ^37^ ^]^ Inductively coupled plasma‐optical emission spectroscopy (ICP‐OES) showed that the proportions of manganese and phosphorus in the MnP were 0.35 and 0.25, respectively (Figure S4, Supporting Information). MnP can be polymerized with organic polymers through hydrogen bonds between the inorganic phosphate and the organic NH_2_ groups, triggering organic–inorganic copolymerization to form the homogenous structure within the composite. Organic–inorganic polymers enhance the loading efficiency and stability of MnP, thereby minimizing their toxicity to cells and tissues.

A schematic illustration of the synthesis of PC6AB polymers, including hydrophilic block and ionizable intelligent recognition block, is shown in Figure S5, Supporting Information. PC6AB was synthesized with poly(ethylene oxide) (PEO) segments and three methacrylate monomers containing C6A, Bn, and aminoethyl (AMA).^[^ ^29^, ^38^, ^39^ ^]^ The incorporated aminoethyl methacrylate (AMA‐MA) where the free amino groups were conjugated to MnP through hydrogen bond or Cy5 through activated N‐hydroxyl succinimidyl (NHS) esters, thereby serving as imaging beacon to investigate optical magnetic dual‐mode imaging. Generally, the hybridization of MnP and PC6AB (PC6AB‐MnP) was achieved by adding MnP to the solution of PC6AB in dichloromethane/ethanol (1:1, v/v, Figure 1e).^[^ ^35^ ^]^ The weight averaged molecular weights (Mw) of PC6AB‐MnP was determined to be 25.1 kDa via gel permeation chromatography (GPC). ATR‐FTIR revealed that the characteristic peak of P‐O stretching vibration in MnP was at 879 cm^−1^, while the peak shifted to 896 cm^−1^ in PC6AB conjugated with MnP (PC6AB‐MnP), indicating the formation of hydrogen bonds between PC6AB and MnP (Figure 1f). The peak was relatively weak due to the small quantity of MnP in the sample. To further confirm the synthesis, Raman spectroscopy was conducted on PC6AB, MnP, and PC6AB‐MnP. The results showed a shift in the P═O stretching band from 1371 cm⁻¹ (MnP) to 1383 cm⁻¹ (PC6AB‐MnP), providing further evidence of the hydrogen‐bond‐mediated interaction between MnP and PC6AB (Figure 1g). The slight shift in the characteristic peak may be attributed to the relatively low content of MnP in PC6AB‐MnP. The powder XRD patterns showed that MnP was amorphous manganese phosphate and PC6AB was a crystalline polymer, indicating the formation of inorganic ionic polymers (Figure 1h). Differential scanning calorimetry (DSC) analysis indicated that there was a difference in the endothermic peak between PC6AB‐MnP and PC6AB, indicating the formation of a new coordination complex between MnP and PC6AB (Figure 1i). Scanning electron microscopy (SEM) was used to monitor the surface morphology of MnP, PC6AB, and PC6AB‐MnP (Figure 1j). The results revealed that MnP displayed a structure consisting of aggregated spherical metal ions, while the organic polymer (PC6AB) exhibited a compact and continuous structure. The surface of PC6AB‐MnP displayed protrusions formed by metal ions, showing the fusion of inorganic oligomers and organic polymer. In specific, the stability of PC6AB‐MnP can be attributed to the formation of hydrogen bonds between organic amino groups and inorganic phosphate groups.

The PC6AB polymers conjugated with MnP and Cy5 were then used to prepare two nanoparticles through self‐assembly: 1) NP: pure PC6AB polymers; and 2) NP‐MnP: PC6AB polymers conjugated with MnP. ICP‐OES was employed to determine the encapsulation efficiency of MnP in the NP‐MnP, which was found to be 86.1%. This high efficiency was attributed to the covalent interaction between the active amino groups in PC6AB and MnP. Dynamic light scattering (DLS) results indicated the hydrodynamic diameter of MnP was 135.2 nm, that of NP was 90.0 nm and that of NP‐MnP was 102.8 nm, with polydispersity indexes of 0.128, 0.141, and 0.111, respectively (Figure S6, Supporting Information). Furthermore, MnP had negative charge (zeta potential ‐34.9 mV). In comparison, NP and NP‐MnP showed positive charge, with zeta‐potential of 30.5 and 31.4 mV, respectively (Figure 1k). The elemental mapping confirmed the presence of manganese, nitrogen, and oxygen elements in NP‐MnP (Figure 1l). Although the element of nitrogen was present in MnP terminated with TEA, its abundance was significantly lower than that of manganese. The elemental mapping results showed that the abundance of nitrogen exceeded that of manganese, which was attributed to the element nitrogen in PC6AB, further confirming the structure within the organic–inorganic copolymer. TEM results showed that NP‐MnP and NP both had spherical morphology at pH 7.4. In comparison, at pH 6.8, ammonium groups of copolymer become highly protonated and showed aqueous solubility as cationic unimer, leading to the degradation of micelles (Figure S7, Supporting Information). Homo‐fluorescence resonance energy transfer (homoFRET)‐induced fluorescence quenching was responsible for complete silencing of the fluorophores Cy5 in the nanoparticle state. The degradation of nanoparticles at low pH leaded to a marked increase in fluorescence emission. NIRI of nanoparticles solutions at different pH values illustrated a sharp fluorescence transition (Figure 1m). Normalized fluorescence intensity as a function of pH showed the fluorescence transition pH (pHt) at 6.80 (Figure S8, Supporting Information). Moreover, manganese has the potential to be the agents for T1‐weighted MRI. To evaluate the MRI sensitivity of MnP, T1‐weighted image of MnP at different concentrations was measured using a 3T magnetic resonance scanner, revealing that the optimal imaging concentration of MnP is approximately 0.25–1.00 mm (Figure 1n). At lower concentrations, the MnP concentration was low enough for its paramagnetic properties to enhance the interaction with water, significantly shortening the T1 relaxation time and thereby enhancing the T1 signal. However, as the concentration increased beyond 0.25 mm, the system began to experience saturation effects, causing diminishing returns in the shortening of the T1 relaxation time. Figure 1o shows that the MRI of MnP‐NP was pH‐dependent, with signal intensity increasing as the pH decreased, which was attributed to the release of MnP from the hydrophobic core of nanoparticles. However, under acidic conditions, nanoparticle depolymerization increased the contact between manganese and water molecules, exhibiting increased relaxation and contrast amplification. The acid‐responsive properties of the nanoparticles were further confirmed by in vitro MRI and NIRI studies. MnP‐NP can be used as acid enhanced fluorescent/magnetic imaging probes for diseases with responsive pathological conditions to offer remarkable therapeutic results and imaging guidance.

### 2.2 pH‐Dependent Membranolytic Selectivity of Nanoagonists

Previous studies have indicated that PC6AB could enable selective plasma membrane rupture in the acidic TME with minimal toxicity in normal tissues.^[^ ^29^ ^]^ In physiological pH environments (pH > 6.8), PC6AB self‐assembles into neutral nanoparticles, and the membranolytic blocks are shielded by PEG shells in a compact core, resulting in minimal membrane dissolution activity (“OFF” state). At the acidic TME, NP‐MnP activates the membranolytic blocks through a rapid transition to the protonated state. Protonated block copolymers facilitate the binding of hydrophobic domains to negatively charged phosphatidylserine, which is highly expressed on the exoplasmic face of tumor cells, through cation‐π interactions. This mechanism inspires the specific recognition of immune cells and tumor cells.^[^ ^40^, ^41^ ^]^ The tumor‐specific membranolytic of NP‐MnP was evaluated in glioma cells GL261 and immune functional microglia BV2 under acidic conditions due to the differences in the structure and composition of cell membranes between the two types of cells.

**Figure 2:**
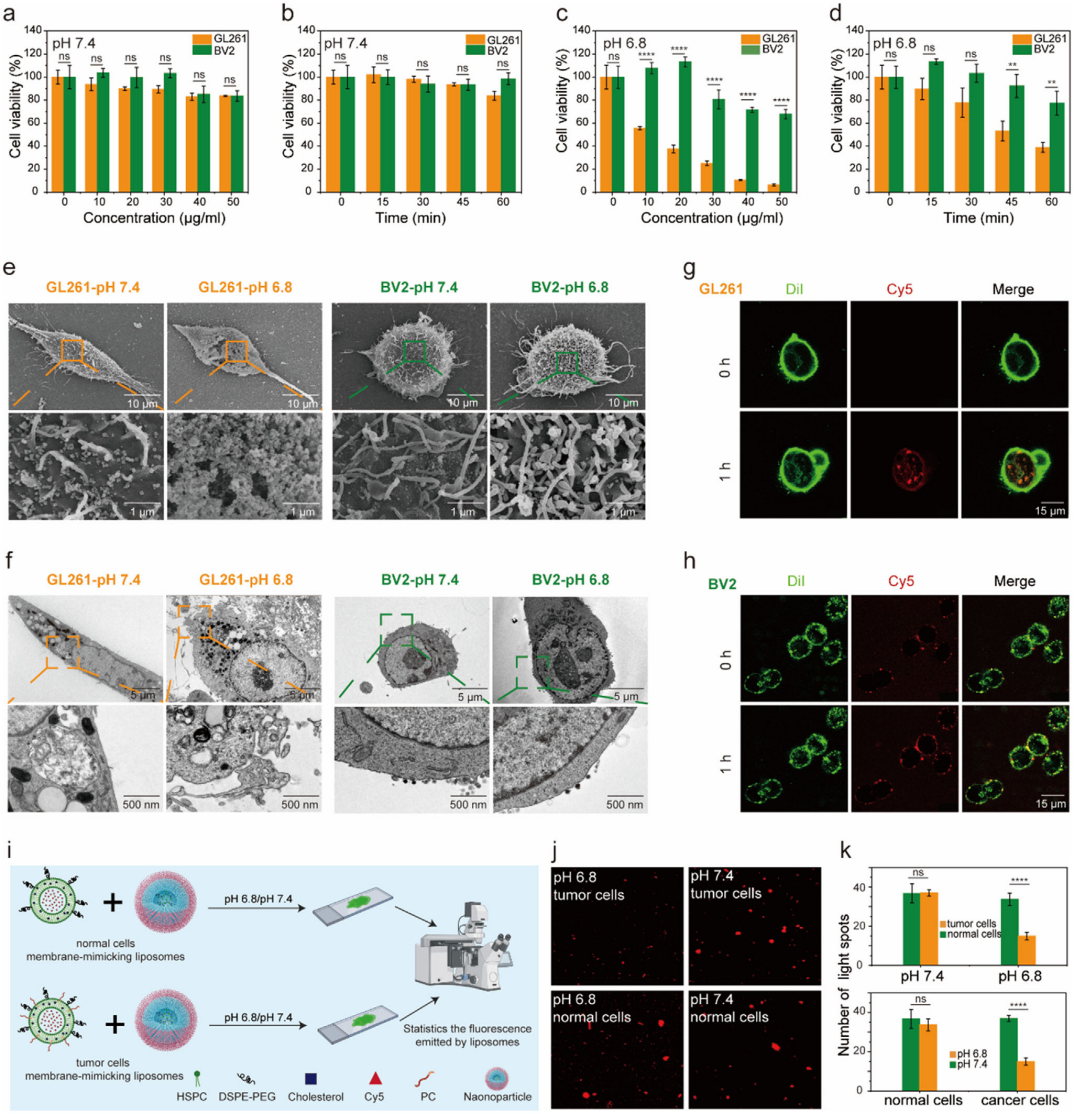
The pH‐dependent membranolytic selectivity of NP‐MnP. a) Concentration‐dependent cytotoxicity of NP‐MnP against GL261 cells and BV2 cells at pH 7.4 after 1 h of incubation. b) Time‐dependent cytotoxicity of NP‐MnP against GL261 cells and BV2 cells at pH 7.4 at a concentration of 25 µg mL^−1^. c) Concentration‐dependent cytotoxicity of NP‐MnP against GL261 cells and BV2 cells at pH 6.8 after 1 h of incubation. d) Time‐dependent cytotoxicity of NP‐MnP against GL261 cells and BV2 cells at pH 6.8 at a concentration of 25 µg mL^−1^. e) SEM images of GL261 cells and BV2 cells after treatment with NP‐MnP (50 µg mL^−1^) at pH 7.4 or 6.8 for 1 h. f) TEM images of GL261 cells and BV2 cells after treatment with NP‐MnP (50 µg mL^−1^) at pH 7.4 or 6.8 for 1 h. g) Images of GL261 cells after treatment with NP‐MnP‐Cy5 (50 µg mL^−1^) at pH 6.8 at different time points (0 and 1 h). h) Images of BV2 cells after treatment with NP‐MnP‐Cy5 (50 µg mL^−1^) at pH 6.8 at different time points (0 and 1 h). i) Schematic diagram of liposome models used to simulate different cell membranes. The membranolytic activity of NP‐MnP was evaluated by observing fluorescence changes in liposomes before and after the addition of NP‐MnP. j) Images of normal cell and tumor cell membrane‐mimicking liposomes after the addition of NP‐MnP at pH 7.4 or 6.8. k) Spot count for each visual field was determined and used for statistical analysis. (n = 3 visual fields). All data were expressed as mean ± SD (n = 3). Statistical analysis was carried out via Student's t‐test or two‐way analysis of variance (ANOVA) method. The significance levels were indicated as follows: ns (no significance), ^**^ *p* < 0.01 and ^****^ *p* < 0.0001. Part **i** was created with BioRender.

We first investigated the selective killing capability of NP‐MnP against BV2 cells and GL261 cells under pH 6.8 or 7.4. At pH 7.4, NP‐MnP showed minimal toxicity against GL261 cells and BV2 cells even at a high concentration or prolonged incubation (**Figure** **2**a,b). At pH 6.8, NP‐MnP exhibited dose‐dependent and time‐dependent toxicity to GL261 cells and BV2 cells. Meanwhile, NP‐MnP displayed a significantly higher cytotoxicity towards GL261 glioma cells compared to BV2 immune cells, providing evidence of selective tumor cell killing without affecting normal immune cells in the TME (Figure 2c,d). To investigate the membranolytic selectivity of NP‐MnP, GL261 cells and BV2 cells were treated with NP‐MnP at 50 µg mL^−1^ for 1 h at pH 6.8 or 7.4. The structure of the cell membrane and the outer morphology of cells were observed using SEM. At pH 7.4, NP‐MnP had no effect on the cell morphology of GL261 cells and BV2 cells. At pH 6.8, there was no significant change on the membranes of BV2 cells incubated with NP‐MnP, however, clear holes appeared on the surface of GL261 cell membranes (Figure 2e). We then used TEM to further observe the cell membrane and other internal components of GL261 cells and BV2 cells after incubation with NP‐MnP. At pH 7.4, NP‐MnP had no effect on the morphology of both cells after 1 h incubation at 50 µg mL^−1^. At pH 6.8, changes in cell membrane morphology and the release of cellular contents caused by cell membrane blebbing were observed in GL261 cells, while BV2 cells did not exhibit the above phenomena (Figure 2f). Electron microscopy data (SEM and TEM) showed clear differences between the effects of NP‐MnP on GL261 and BV2 cells. At pH 6.8, NP‐MnP induced visible membrane damage and cellular content release in GL261 cells, while BV2 cells did not exhibit such changes, indicating the selective disruption of tumor cell membranes. Confocal laser scanning microscopy (CLSM) was used to observe the interaction between NP‐MnP and the cell membrane at pH 6.8. NP‐MnP conjugated with Cy5 is referred to as NP‐MnP‐Cy5 for fluorescence imaging. NP‐MnP‐Cy5 did not affect the cell morphology and membrane integrity of BV2 cells within 1 h. In contrast, NP‐MnP‐Cy5 caused cells to swell, generated bubble‐like herniations, and resulted in the leakage of cellular contents in GL261 cells (Figure 2g,h).

At pH 6.8, the conversion of nanoagonists into cationic particles containing phenyl groups facilitates binding to negatively charged cell membranes. The benzyl groups can penetrate into the membrane and contact with hydrophobic lipid tails through cation‐π interactions, leading to membrane disruption. In contrast to normal cells with neutral phospholipids, cancer cell membranes expose high levels of negatively charged phosphatidylserine outside the plasma membrane. This leads to stronger interactions and membrane splitting effects between NP‐MnP and GL261 cells, promoting membranolytic selectivity. To further validate this, we prepared liposomes mimicking normal cell membranes and liposomes incorporating phosphatidylserine to represent tumor cell membranes. Fluorescent dyes were encapsulated within the liposomes to monitor their membrane integrity. The rupture of the liposome membrane resulted in the disappearance of fluorescent spots, and membrane integrity was assessed by counting the remaining fluorescent spots. We co‐incubated the two types of liposomes with NP‐MnP separately at predefined pH (pH 7.4 or 6.8), and CLSM was used to observe the fluorescence emitted by the liposomes (Figure 2i). At pH 7.4, there was no significant difference in the number of fluorescent spots between the two types of liposomes incubated with NP‐MnP. At pH 6.8, we observed a significant reduction in the number of fluorescent spots in the liposomes simulating tumor cells, indicating dye leakage and membrane rupture. In contrast, the number of fluorescent bright spots in the liposomes simulating normal cells incubated with NP‐MnP showed no significant change at pH 6.8 (Figure 2j,k). These results indicated that the differences in phosphatidylserine content between tumor cells and normal cells led to the membranolytic selectivity of NP‐MnP.

### 2.3 In Vitro Activation of STING Pathway

Immunotherapy has revolutionized cancer treatment in the past decade.^[^ ^10^ ^]^ However, the aggressive brain tumor glioblastoma is highly immunosuppressive due to impaired immune function, such as antigen‐presenting function, cross‐priming, and immune stimulation. Nanoagonists composed of bioactive metal ions manganese and STING agonists represent promising strategies to improve the antitumor efficacy in glioma. Meanwhile, nanoparticles can solve the problems of rapid enzymatic degradation and off‐target toxicity of STING agonists, thereby improving their bioavailability and pharmacological activity.

**Figure 3:**
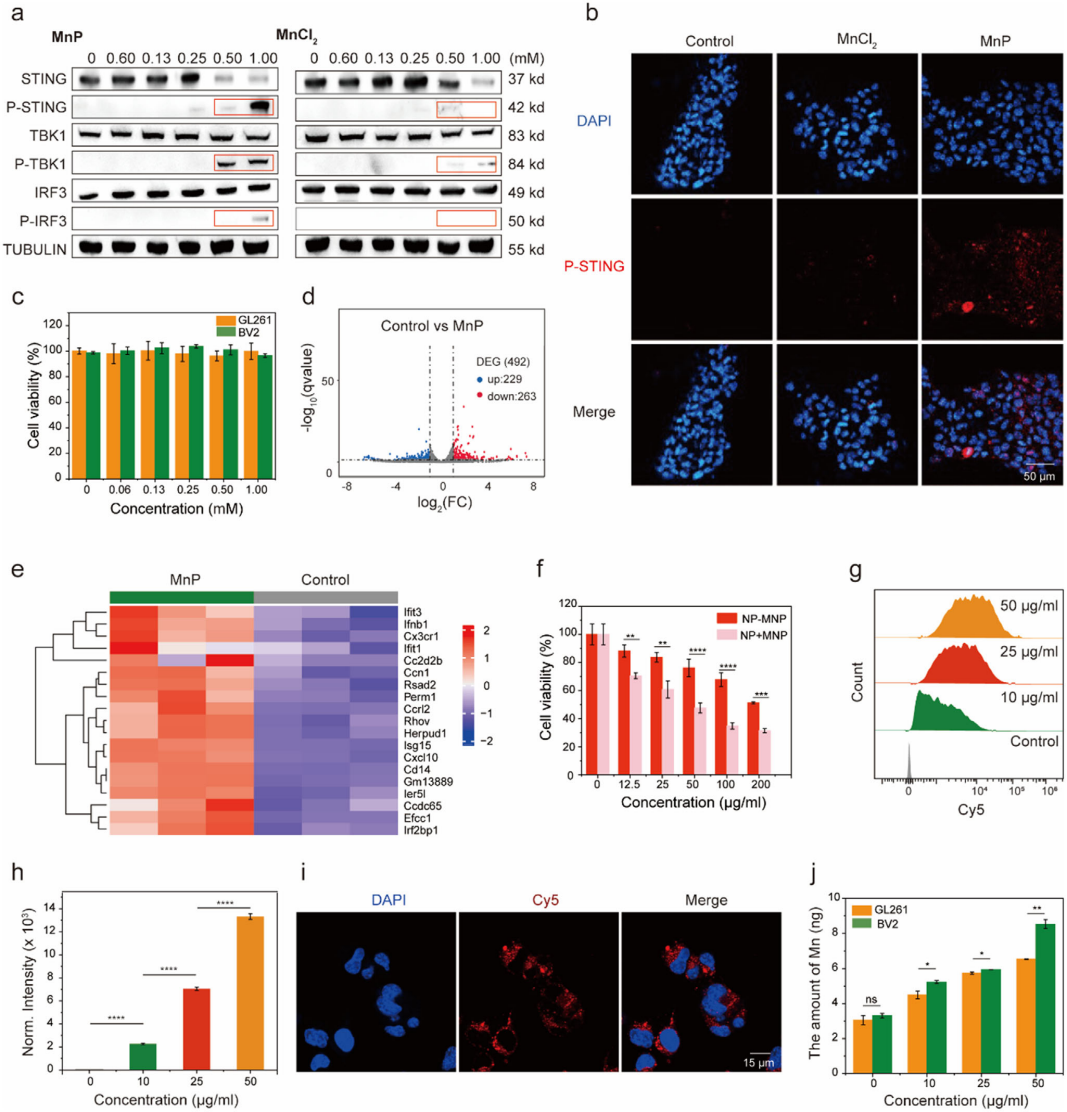
MnP activated the cGAS‐STING pathway, and intracellular uptake of nanoagonists in vitro. a) Concentration‐dependent changes in the expression levels of STING pathway‐related proteins upon treatment with MnCl₂ or MnP, as determined by western blot analysis. b) CLSM images of P‐STING expression following treatments. c) Concentration‐dependent cytotoxicity of MnP against GL261 cells and BV2 cells. The data were expressed as mean ± SD (n = 3). Statistical analysis was carried out via two‐way ANOVA method. The P‐values greater than 0.05 indicated no significant difference. d) Volcano plots displayed the differentially expressed genes between cells treated by PBS and MnP. e) Heat map of gene expression in cells treated with PBS and MnP. f) Concentration‐dependent cytotoxicity of NP‐MnP or NP+MnP at pH 7.4 after 1 h of incubation (NP‐MnP: nanoparticles self‐assembled from co‐polymerized PC6AB‐MnP, NP+MnP: self‐assembled nanoparticles from PC6AB with an equal amount of MnP added). g,h) Representative flow cytometric profiles (g) and semi‐quantification (h) of concentration‐dependent uptake of NP‐MnP‐Cy5 by BV2 cells. i) Representative CLSM images of BV2 cells incubated with NP‐MnP‐Cy5. j) Intracellular manganese (Mn) uptake was measured by ICP‐MS in GL261 and BV2 cells treated with NP‐MnP (10, 25, and 50 µg mL^−1^). All data were expressed as mean ± SD (n = 3). Statistical analysis was carried out via one‐way or two‐way ANOVA method. The significance levels were indicated as follows: ns (no significance), ^*^ *p* < 0.05, ^**^ *p* < 0.01, ^***^ *p* < 0.001, and ^****^ *p* < 0.0001.

Manganese, as an immune adjuvant, is an activator of the cGAS‐STING pathway. In order to compare the effects of MnP and MnCl_2_ on the STING pathway activation, western blot was performed to analyze the expression of associated enzymes in the cells after 24 h of incubation with different concentrations of MnP and MnCl_2_. The results showed that the expression levels of phospho‐TBK1 (P‐TBK1), phospho‐IRF3 (P‐IRF3), and phosphorylated STING (P‐STING) were upregulated following treatment with increasing concentration of manganese. The expression of STING decreased with increasing concentrations of manganese, indicating that the STING protein underwent phosphorylation. Notably, the levels of phosphorylated proteins were even higher when cells were incubated with MnP, suggesting that MnP has a stronger activating effect on STING compared to MnCl_2_ (**Figure** **3**a). The upregulation of P‐TBK1, P‐IRF3, and P‐STING expression in cells was observed using immunofluorescence, with MnP showing stronger activation of the STING pathway than MnCl_2_ (Figure 3b; Figure S9, Supporting Information). We conducted cytotoxicity experiments to assess the effects of manganese on GL261 cells and BV2 cells under the same cell number and MnP concentration conditions as used in the western blot analysis. The results demonstrated that MnP did not exhibit significant toxicity within the effective concentration range (Figure 3c). Previous studies have shown that manganese activates the STING pathway by binding to cGAS, which triggers the production of interferons and thereby activates the immune response.^[^ ^42^ ^]^ To further validate the mechanism by which MnP activated the STING pathway, we conducted genome‐wide RNA‐sequencing analysis of cells treated with MnP. The volcano plot results indicated that there were 492 differentially expressed genes between the control and MnP groups, with 229 genes upregulated and 263 genes downregulated (Figure 3d). Figure 3e shows a heat map of the expression pattern of differentially expressed genes between the cells treated with PBS and MnP. Compared to the cells treated with PBS, the cells treated with MnP exhibited upregulation of genes such as Ifnb1, Isg15, and Cxcl10. Enrichment analysis by Gene Ontology (GO) further revealed that compared to PBS, MnP mainly affected the expression of genes in response to interferon‐beta (IFN‐β), interferon‐gamma production (IFN‐γ), and positive regulation of T cell‐mediated immunity (Figure S10, Supporting Information). These results indicated that MnP stimulated immune activation by inducing the production of interferons.

Previous study has reported Mn^2^⁺ can stimulate immune responses in a phosphate solution, whereas Mn^2^⁺ in normal saline does not have the same effect. Moreover, Mn^2+^ in phosphate‐buffered solution tends to aggregate and form precipitates, resulting in a loss of its adjuvant activity.^[^ ^43^ ^]^ To overcome this challenge, MnP was synthesized using Mn^2^⁺, phosphate, and TEA. The inclusion of TEA stabilized manganese phosphate, enhancing the effectiveness of MnP in activating the STING pathway. Additionally, the nanoparticle structure of MnP enhances the efficiency of cell uptake, resulting in a more potent activation of the STING pathway compared to MnCl_2_. We analyzed the toxicity of MnP and MnCl_2_ on cells by using the same concentration in the western blot analysis but with a reduced cell amount. Our research showed that there was no significant difference in the cytotoxicity between MnP and MnCl_2_ (Figure S11, Supporting Information). We analyzed the cytotoxicity of nanoparticles self‐assembled from co‐polymerized MnP and nanoparticles with an equal amount of MnP added, and the results showed that nanoparticles with added MnP exhibited significantly higher cytotoxicity. (Figure 3f). Notably, MnP can be polymerized with organic polymers to form nanoparticles that are encapsulated in a hydrophobic core under physiological environment, exhibiting low toxicity.

The intracellular delivery and immune‐stimulatory effect of nanoagonists were investigated on BV2 microglia cells, which function as immunoreactive cells in the nervous system. BV2 cells were incubated with NP‐MnP‐Cy5 and the internalization into the cells was analyzed by flow cytometry. The results showed that as the concentration of NP‐MnP‐Cy5 increased, the fluorescence intensity within the BV2 cells gradually increased (Figure 3g,h). These findings indicated that the uptake of NP‐MnP‐Cy5 by the cells was concentration‐dependent. Flow cytometry was employed to analyze the impact of various uptake inhibitors on cell uptake. The results showed that glucose and nystatin exhibited significant inhibition of cell uptake, indicating that the primary mechanism of NP‐MnP‐Cy5 uptake by cells occurred via caveolin‐pathways and energy‐dependent endocytosis (Figure S12, Supporting Information). CLSM was employed to monitor cell uptake, revealing substantial intracellular internalization of NP‐MnP‐Cy5 (Figure 3i). Inductively coupled plasma mass spectrometry (ICP‐MS) was used to measure the uptake of manganese from NP‐MnP by BV2 cells and GL261 cells. The results indicated that BV2 cells took up a greater amount of manganese compared to GL261 cells, with this trend becoming more pronounced over time (Figure 3j). We analyzed that the higher manganese content in BV2 cells was not due to an inherent specificity of MnP for immune cells over tumor cells. Instead, the preferential uptake of MnP by immune cells occurred as a result of tumor cell death induced by PC6AB treatment. While PC6AB did not cause damage to immune cells, it induced the death of tumor cells. Following tumor cell death in vivo, immune cells were recruited to the site of the tumor, where they took up MnP to enhance immune activation. Additionally, the uptake of MnP by tumor cells can also trigger the immune activation response. The impact of MnP on the expression levels of STING activation‐related proteins upon treatment of GL261 cells with MnP was investigated using western blot analysis (Figure S13a, Supporting Information). Consistent with results observed in immune cells, the protein level of P‐STING was upregulated in response to treatment with higher MnP concentrations. Immunofluorescence analysis further demonstrated the upregulated expression of P‐STING proteins in GL261 cells (Figure S13b, Supporting Information). These results indicated that MnP also activated the STING pathway in tumor cells.

**Figure 4:**
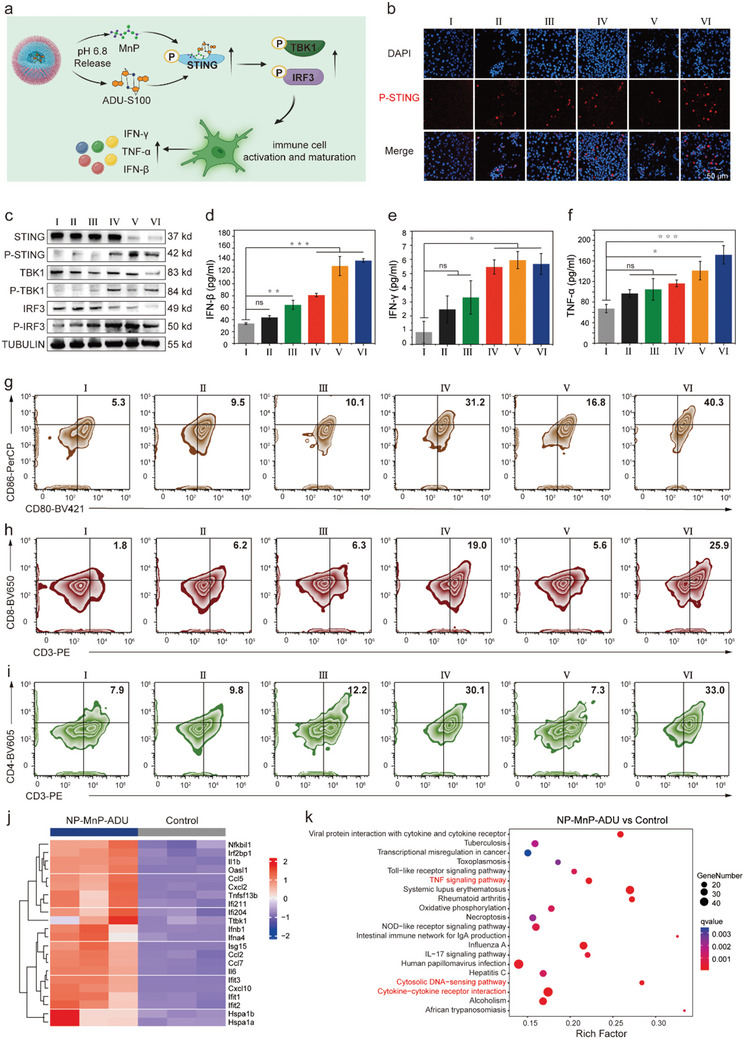
Nanoagonists stimulated immune activation mediated by the cGAS‐STING pathway in vitro. a) Schematic illustration of the mechanism by which nanoagonists activate the STING pathway. b) CLSM images of P‐STING immunofluorescence in BV2 cells after different treatments. c) Western blot analysis of the expression levels of STING, P‐STING, TBK1, P‐TBK1, IRF3, and P‐IRF3 in BV2 cells treated with supernatants from GL261 cells after different treatments. d–f) Secretion levels of immune‐stimulatory cytokines including IFN‐β (d), IFN‐γ (e), and TNF‐α (f) in the supernatant from the above co‐culture system with different treatments. g–i) Representative flow cytometry images of the expression levels of CD80^+^/CD86^+^ (g), CD3^+^/CD4^+^ (h), and CD3^+^/CD8^+^ (i) in BV2 cells co‐incubated with GL261 cells after different treatments. Different treatments including: (I) Control, (II) NP, (III) MnP, (IV) NP‐MnP, (V) ADU, and (VI) NP‐MnP‐ADU. All flow cytometry experiments were repeated three times independently with similar results. j) Heat map of gene expression in cells treated with NP‐MnP‐ADU and PBS. k) KEGG analysis of differentially expressed genes between cells treated with NP‐MnP‐ADU and PBS. All data were expressed as mean ± SD (n = 3). Statistical analysis was carried out via one‐way ANOVA method. The significance levels were indicated as follows: ns (no significance), ^*^ *p* < 0.05, ^**^ *p* < 0.01, and ^***^ *p* < 0.001. Part **a** were created with BioRender.

The organic–inorganic copolymerization was used to prepare nanoparticles containing manganese and STING agonists ADU‐S100 (NP‐MnP‐ADU), which form complexes with cGAS in immune cells of the nervous system to generate strong anticancer immune responses (**Figure** **4**a). To test the immune cell activation efficiency of nanoagonists, the supernatant from nanoagonist‐treated GL261 tumor cells was extracted and utilized for BV2 microglia cells. Using immunofluorescence CLSM, the upregulated expression of P‐TBK1, P‐IRF3, and P‐STING inside the cells co‐cultured with NP‐MnP‐ADU was visualized (Figure 4b; Figure S14, Supporting Information). P‐IRF3 typically translocates from the cytoplasm to the nucleus, where it activates the transcription of immune response‐related genes. To enhance clarity, we included partial zoom‐in images of the merged panels in Figures S9 and S14 (Supporting Information), which allowed for a more detailed observation of P‐IRF3 localization. Immunofluorescence results clearly demonstrated that P‐IRF3 was expressed in the nuclei of cells treated with different conditions, reflecting the activation of the immune response (Figure S15, Supporting Information). Western blot data showed that the expression levels of major components in cGAS‐STING pathway including P‐STING, P‐IRF3, and P‐TBK1 were upregulated in NP‐MnP‐ADU‐treated BV2 cells (Figure 4c). Due to the disassembly of nanoagonists in the TME, PC6AB specifically lysed the GL261 cell membrane to produce immunogenicity, which worked together with MnP and ADU to activate the STING pathway of BV2 cells, leading to a significant immune activation effect of NP‐MnP‐ADU on BV2 cells.

To test that nanoagonists can enhance tumor‐specific immunity, we designed a co‐culture system of GL261 tumor cells and BV2 immune cells to mimic the TME. Firstly, the secretion of IFN‐β, IFN‐γ, and tumor necrosis factor alpha (TNF‐α) were studied by an ELISA assay. Compared to the control group, upon treatment with NP‐MnP‐ADU, the levels of IFN‐β were found to be approximately 4.2‐fold augmented, the levels of IFN‐γ 6.6‐fold enhanced and the levels of TNF‐α 2.5‐fold increased, indicating the activation of the STING pathway and the release of interferons and inflammatory factors (Figure 4d,f). BV2 cells exhibit functions analogous to those of dendritic cells in the nervous system. To evaluate the immune activation effect, we labeled BV2 cells with CD11C (a marker of microglial cells) and assessed co‐stimulatory molecules (CD80 and CD86).^[^ ^44^, ^45^, ^46^ ^]^ As shown in Figure 4g, NP‐MnP‐ADU treatment increased the population of CD80^+^/CD86^+^ within BV2 cells, which was 7.6‐fold higher than the control group (Figure 4g). These results confirmed that ADU release and MnP stimulation enhanced BV2 cell maturation. Recent studies have shown that BV2 cells are involved in neuroinflammation and immune regulation, exhibiting context‐dependent plasticity when facing induced stimuli.^[^ ^47^, ^48^ ^]^ As a result of enhanced immune function in the co‐incubation system, the amount of CD3^+^/CD4^+^ T cells increased by 25.1%, along with a 24.1% increase in CD3^+^/CD8^+^ cells compared to the control group (Figure 4h,i). This suggests that BV2 cells are adopting an antigen‐presenting cell‐like phenotype, capable of interacting with T cells. The phenomenon highlights the complexity of immune cell marker expression under pathological stimuli. Overall, NP‐MnP‐ADU enhanced the magnitude of the STING‐mediated immune response through a dual mechanism of activation of both MnP and ADU.

To investigate the mechanism underlying the antitumor immune response, we conducted RNA sequencing on cells treated with NP‐MnP‐ADU. Compared to cells treated with PBS, 1508 genes were upregulated (red dots) and 757 genes were downregulated (blue dots) in cells treated with NP‐MnP‐ADU (Figure S16, Supporting Information). RNA sequencing results showed that Ifnb1, Cxcl2, and Ccl5 genes were upregulated in cells treated with NP‐MnP‐ADU (Figure 4j). These findings suggest that NP‐MnP‐ADU induces the release of cytokines and interferons, which play crucial roles in activating immune responses. In addition, we performed GO analysis, which indicated that NP‐MnP‐ADU treatment affected gene expression in pathways related to the response to IFN‐β, regulation of cytokine production, and immune response (Figure S17, Supporting Information). Through Kyoto Encyclopedia of Genes and Genomes (KEGG) enrichment analysis, we identified several key biological pathways that are significantly altered in our experimental conditions. These pathways included TNF signaling pathway, cytosolic DNA sensing pathway, and cytokine‐cytokine receptor interaction (Figure 4k). This suggested that the cytosolic DNA‐sensing pathway could indeed be activated by NP‐MnP‐ADU, which was required to activate the STING pathway. The STING pathway induced the production of cytokines that activated cytokine receptors, further amplifying immune responses. In conclusion, NP‐MnP‐ADU activated the STING pathway through the DNA sensing mechanism, thereby inducing the release of cytokines and interferons to exert antitumor immune effects.

### 2.4 In Vivo Antitumor Effects

**Figure 5:**
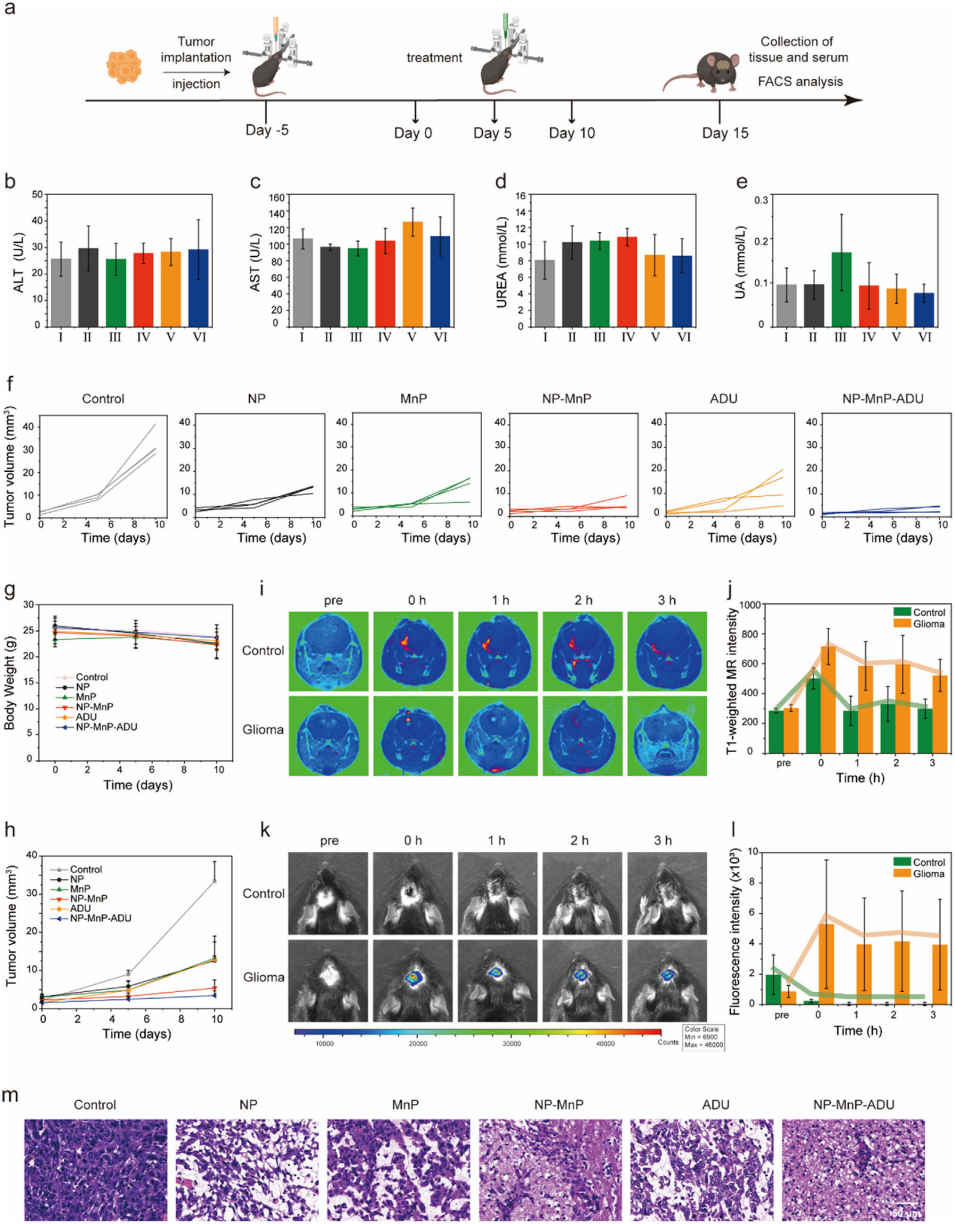
Local intratumoural administration of nanoagonists eliminated established tumors. a) Schematic illustration of the timeline for the establishment and treatment of the mouse model. b–e) Concentrations of ALT (b), AST (c), UREA (d), and UA (e) in the serum 48 h after different treatments to evaluate their hepatotoxicity and nephrotoxicity. (I) Control, (II) NP, (III) MnP, (IV) NP‐MnP, (V) ADU, and (VI) NP‐MnP‐ADU. All data were expressed as mean ± SD (n = 4). Statistical analysis was carried out via one‐way ANOVA method. The P‐values greater than 0.05 indicated no significant difference. f) Tumor growth inhibition curves of mice after different treatments. g) Body weights of mice over the treatment period. h) Tumor volume of tumor‐bearing mice was monitored after different treatments. i) Real‐time MRI of healthy and tumor‐bearing mice after single dose administration of NP‐MnP‐Cy5 via CED. j) The MRI signal intensities changing after single dose administration of NP‐MnP‐Cy5 via CED. k) Real‐time fluorescence images of healthy and tumor‐bearing mice after single dose administration of NP‐MnP‐Cy5 via CED. l) The fluorescence signal intensities changing after single dose administration of NP‐MnP‐Cy5 via CED. m) H&E staining results of brain tissue samples after different treatments. All data were expressed as mean ± SD (n = 4). Part **a** was created with BioRender.

Based on the promising in vitro anticancer effects, the antitumor efficacy of nanoagonists was further studied in the GL261 in situ glioma mouse model. NP‐MnP‐ADU consisted of membranolytic block copolymer PC6AB coupled with MnP, which encapsulated the STING agonist ADU. PC6AB has membranolytic activity, while ADU and immune adjuvant MnP modulate the immune microenvironment, resulting in enhanced immune activation and antitumor effects. To test the therapeutic efficacy of the cooperative cGAS‐STING nanoagonists, we first established an animal model by implanting GL261 cells into the caudate nucleus of mice. Five days after tumor engraftment, mice received the treatments of PBS, NP, MnP, NP‐MnP, ADU, or NP‐MnP‐ADU every 5 d for a total of 15 d (**Figure** **5**a). In published studies, nanoparticles were generally administered through tail vein at doses ranging from 25 to 50 mg kg^−1^ for tumor treatment.^[^ ^49^ ^]^ CED method was used to deliver nanoagonists at a dose of 0.75 mg kg^−1^, which effectively crossed the blood‐brain barrier and showed high biocompatibility.^[^ ^50^ ^]^ We also comprehensively investigated the toxicity of the nanoagonists on GL261 tumor‐bearing C57BL/6 mouse model after systemic administration. The histological analysis of the major organs (heart, spleen, lungs, and kidneys) stained with hematoxylin and eosin (H&E) did not reveal any apparent morphological changes, indicating that nanoagonists did not cause acute toxicity (Figure S18, Supporting Information). The abnormalities observed in the control, NP‐MnP, and NP‐MnP‐ADU groups were not caused by the drug treatment, but rather resulted from edema during the experimental procedures. We also monitored key liver indices (alanine aminotransferase, ALT and aspartate aminotransferase, AST) and kidney indices (urea and uric acid, UA) after different treatments and found that all these parameters were within normal range with no significant changes, indicating that nanoagonists did not induce liver or renal toxicity (Figure 5b–e). Negligible changes in the body weight of the mice in different groups were observed throughout the study (Figure 5g). Studies have shown that CED treatment for glioblastoma is safer than intraperitoneal injection.^[^ ^51^ ^]^ The results of this study indicated that no observable toxicity was detected in the mice treated with CED.

The antitumor efficacy of NP‐MnP‐ADU was then investigated in mice bearing GL261 tumors. The tumors of mice treated with PBS showed exponential growth, and the tumor size increased to around 32.6 mm^3^ after 20 d. ADU, a well‐synthetic small molecule agonist, is considered a promising anticancer agent with remarkable preclinical efficacy in some tumor models. Initial research also suggests its potential for tumor treatment through STING activation in the brain.^[^ ^28^, ^30^ ^]^ NP, ADU, and MnP treatments moderately inhibited tumor progression with the average tumor size being around 13 mm^3^. There were no significant differences in tumor size among the mice treated with these three drugs (Figure 5f). In comparison, the treatment of NP‐MnP‐ADU, which combined STING activation and membrane rupture showed the most significant tumor inhibition effect with an average final tumor size of around 3.42 mm^3^ (Figure 5h). After the treatments, the tumor tissues from mice were collected for histological examination. H&E staining showed clear nuclear fragmentation and nuclear lysis of the cancer cells during the treatment with the nanoagonists (Figure 5m). Overall, these findings demonstrated the strong antitumor efficacy of NP‐MnP‐ADU by integrating chemotherapy and immunotherapy.

To monitor the distribution of NP‐MnP‐ADU and its pH‐responsive imaging in tumor tissue, we evaluated T1‐weighted MRI and fluorescence images in glioma mice after a single dose treatment of NP‐MnP‐Cy5 (3 µL) via CED. In T1‐weighted MRI, a rapid contrast enhancement in tumors of GL261‐tumor model mice was achieved after the administration of NP‐MnP‐Cy5 (Figure 5i), while health mice did not show any enhancement in T1 contrast, indicating that the pH‐triggered release of manganese was critical for tumor probing. These results proved that the release of manganese from NP‐MnP‐Cy5 in the acidic TME improved imaging contrast, and the binding of manganese to proteins further amplified the signal. After injecting NP‐MnP‐Cy5 the contrast rapidly increased within 10 min and maintained for approximately 3 h with sufficient tumor specificity, whereas the signal remained almost unchanged in healthy mice (Figure 5j). We also observed a gradual decrease in the contrast over time, indicating that the NP‐MnP‐Cy5 underwent metabolism and elimination through the brain ECS. Furthermore, fluorescence imaging was carried out to track the in vivo behaviors of NP‐MnP‐ADU after injection into tumor model via CED. Strong intensity of fluorescence signals was mainly shown for NP‐MnP‐Cy5 in the tumor site, which was significantly higher than the fluorescence signal of the control group. In contrast, no obvious fluorescence signal appeared in the health mouse for NP‐MnP‐Cy5 even after 3 h (Figure 5k,l). After 48 h, the mice were euthanized and the excised main organs (liver, spleen, lung, heart, and kidney) were imaged to analyze the metabolic pathways of NP‐MnP‐Cy5. The results showed strong fluorescence signals in the liver and kidneys, indicating that NP‐MnP‐Cy5 was mainly metabolized by the liver and kidneys after administration via the brain ECS (Figure S19, Supporting Information).

### 2.5 NP‐MnP‐ADU Activates Antitumor Immunity

**Figure 6:**
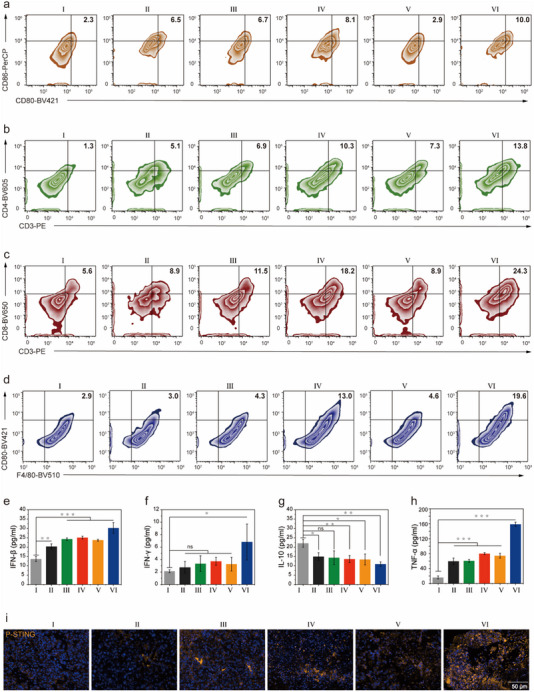
Nanoagonists activated the cGAS‐STING pathway to promote cytokines release and immune system activation. a–d) Representative flow cytometry images of the expression levels of CD80^+^/CD86^+^, CD3^+^/CD4^+^, CD3^+^/CD8^+^ and F4/80/CD80^+^ in GL261 tumor tissues after treatments in vivo. e–h) Secretion levels of IFN‐β, IFN‐γ, IL‐10, and TNF‐α in the serum after different treatments. i) Immunofluorescence analysis of the expression level of P‐STING in the tumor after different treatments, Yellow: P‐STING, Blue: nuclei. Different treatments including: (I) Control, (II) NP, (III) MnP, (IV) NP‐MnP, (V) ADU, and (VI) NP‐MnP‐ADU. All flow cytometry experiments were repeated 3 times independently with similar results. All data were expressed as mean ± SD (n = 3). Statistical analysis was carried out via one‐way ANOVA method. The significance levels were indicated as follows: ^*^ *p* < 0.05, ^**^ *p* < 0.01, and ^***^ *p* < 0.001.

Inspired by the superior antitumor immune response of NP‐MnP‐ADU, we conducted the analysis of the blood, tumors, and spleens of the animals receiving different treatments. The immune responses in tumors and spleens were analyzed with flow cytometry. The results showed that the expression of CD80^+^/CD86^+^ in the cells of GL261‐tumor‐bearing mice treated with NP‐MnP‐ADU was 4.3‐fold higher than the control group, indicating that NP‐MnP‐ADU can promote the maturation of immune cells (**Figure** **6**a). Meanwhile, the ratio of mature dendritic cells in the spleen was significantly higher in mice treated with NP‐MnP‐ADU than the control group, due to the activated antitumor immunity and improved homing of immune cells (Figure S20a, Supporting Information). Additionally, the activated T cell populations (CD4^+^/CD8^+^ T cells) in tumor tissues and spleens after different treatments were examined. The population of CD4^+^ T cells in the tumors after NP‐MnP‐ADU treatment was 10.6, 2.7, 2.0, 1.3, and 1.9‐fold higher than those in mice treated with PBS, NP, MnP, NP‐MnP, and ADU, respectively. The population of tumor‐infiltrating CD8^+^ T cells also increased after the treatment of NP‐MnP‐ADU (Figure 6b,c). Consistently, NP‐MnP‐ADU treatment induced higher infiltration of CD4^+^ and CD8^+^ T cells in the spleen than the control group, suggesting that nanoagonists can promote immune cell maturation and T cell activation (Figure S20b,c, Supporting Information). Moreover, we observed an increase in the pro‐inflammatory M1 macrophages phenotype in mouse tumors treated with NP‐MnP‐ADU (Figure 6d). M1 macrophages have immune‐stimulatory functions, suggesting that NP‐MnP‐ADU can mediate the repolarization of M2 macrophages to the M1 phenotype, thereby alleviating immune suppression in the ECS and enhancing T cell‐mediated antitumor efficacy. Overall, these findings demonstrated that NP‐MnP‐ADU was capable of promoting immune cell maturation, inducing the production of active T cells, and reshaping the TME into an immunosupportive state to activate tumor immune responses.

Additionally, treating tumors with NP‐MnP‐ADU significantly increased the levels of IFN‐β, IFN‐γ, and TNF‐α in the serum, which were approximately 2.2, 3.2, and 9.7‐fold higher than those in the control group, and the Interleukin 10 (IL‐10) in serum decreased by 50% compared to the control group (Figure 6e–h). These results indicated pro‐inflammatory cytokines mediated an efficient antitumor immune response. We further observed the overexpression of P‐STING, P‐TBK1, and P‐IRF3 proteins in tumor tissues via immunofluorescence imaging, indicating that the immune response in the animal model was triggered through activation of the cGAS‐STING pathway (Figure 6i; Figure S21, Supporting Information). Taken together, the potent antitumor immune responses of NP‐MnP‐ADU were mediated through multiple mechanisms. On one hand, NP‐MnP‐AUD activated the cGAS‐STING pathway, stimulating T lymphocytes and promoting the production of interferons and other pro‐inflammatory factors. On the other hand, NP‐MnP‐ADU alleviated immunosuppression in the TME by increasing the proportion of M1 macrophages, thereby enhancing the responses of immune cells and inhibiting tumor growth.

## 3 Conclusion

This study polymerized inorganic polymers with organic polymers to activate the STING pathway and reverse immunosuppression, demonstrating no significant toxicity in vivo. The organic–inorganic hybrid polymers approach expands the application of metal ions in the biomedical field, addressing issues related to the short circulation time and high acute toxicity of metal ions, while providing a promising platform for future processes and applications. In vivo antitumor immune response results demonstrated that NP‐MnP induced a stronger immune activation effect compared to MnP, confirming the synergistic effect of NP and MnP in stimulating immune cell maturation. The synergistic effects of the NP‐MnP may be attributed to the membranolytic activity of NP, which induces immunogenicity and releases abundant tumor‐derived double‐stranded DNA (dsDNA) into the TME, enhancing the immunogenicity of tumor models and offering new opportunities for clinical immunotherapy.

PC6AB demonstrates strong membranolytic activity due to its benzyl groups, which form non‐covalent aromatic interactions with cell membranes, inducing local membrane curvature damage and disrupting membrane integrity. In contrast to normal cells, which possess neutral phospholipids, tumor cell membranes exhibit higher levels of negatively charged phosphatidylserine on their surface, increasing the affinity of PC6AB for the membrane and promoting membranolytic selectivity. Furthermore, CED, a technique widely tested in clinical trials, can bypass the blood‐brain barrier, delivering drugs directly to the target region. This enhances drug distribution and minimizes localization in healthy tissues. The clinical promise for CED agents extends to the adjuvant treatment of recurrent and inoperable deep brain tumors, improving survival or providing symptomatic relief.^[^ ^52^ ^]^ Based on the superior antitumor effects of intratumoral delivery of nanovaccines compared with subcutaneous delivery, this study combines nanoagonists with CED for glioma treatment, enhancing immune activation, reducing dosage and alleviating neurotoxicity and systemic toxicity in vivo. Although the efficacy of nanoagonists in mouse models is promising, several challenges remain, including the need to enhance PC6AB selectivity through ligand modification, optimize the preparation process, and evaluate the safety and efficacy of these materials in primate models.

In conclusion, we have developed a multifunctional nanoagonist that simultaneously leads to cancer cell membrane rupture and STING pathway activation. This nanoagonist activated the STING pathway, alleviated tumor immune suppression, promoted the infiltration and activation of immune cells, and enhanced the release of inflammatory factors, thereby potently activating antitumor immunity for the treatment of glioma. We anticipate that spatiotemporal coordination of immunotherapy and chemotherapy could be extended to other cancer immunotherapies, providing a potential strategy to overcome immune suppression in solid tumors.

## 4 Experimental Section

### Animals

Male C57BL/6 mice (6–8 weeks, 20–25 g) were purchased from the Peking University Health Science Center and all mice were kept in a pathogen‐free animal facility specific to the Department of Laboratory Animal Sciences. All animal experimental protocols were conducted in accordance with the Animal Management Rules of the Ministry of Health of the People's Republic of China and were approved by the Institutional Animal Care and Use Committee (IACUC) of Peking University Health Science Center (Animal Protocol Approval Number: BCJE0156).

### Statistical Analysis

All measurements were performed on three or more independent replicates from separate experiments. The number of replicates performed was indicated in each figure legend. Results were expressed as mean ± standard deviation (SD). All statistical data were processed in Origin 2022. Two groups were compared using two‐sided Student's *t*‐tests, while comparisons among three or more groups were performed using one‐way or two‐way ANOVA, depending on the experimental design. A P‐value less than 0.05 was considered statistically significant. The significance levels were indicated as follows: ns (no significance), ^*^ *p* < 0.05, ^**^ *p* < 0.01, ^***^ *p* < 0.001, and ^****^ *p* < 0.0001.

An additional experimental section is included in the supporting information.

## Conflict of Interest

The authors declare no conflict of interest.

## Supporting information



Supporting Information

## Data Availability

The data that support the findings of this study are available from the corresponding author upon reasonable request.
