# Supplementary material for: Smart Organic–Inorganic Copolymer Nanoparticles Distinguish Between Microglia and Cancer Cells for Synergistic Immunotherapy in Glioma
Source: Adv Sci (Weinh). 2025 Apr 29;12(25):2500882. doi: 10.1002/advs.202500882 (PMC12224928; doi:10.1002/advs.202500882)
Supplement: Supplementary file 1 — Supporting Information [file ADVS-12-2500882-s001.docx]

Supporting Information

**Smart Organic-Inorganic Copolymer Nanoparticles Distinguish between Microglia and Cancer Cells for Synergistic Immunotherapy in Glioma**

*Shiming Zhang^#^, Kun Shang^#^, Lidong Gong, Qian Xie, Jianfei Sun, Meng Xu, Xunbin Wei, Zhaoheng Xie, Xinyu Liu, Hao Tang, Zhengren Xu, Wei Wang, Haihua Xiao*, Zhiqiang Lin*, Hongbin Han**

S. Zhang, M. Xu, X. Wei, Z. Xie, X. Liu

Institute of Medical Technology, Peking University Health Science Center

Beijing 100190, P. R. China

K. Shang

Department of Nuclear Medicine, Peking University People's Hospital

Beijing 100190, P. R. China

L. Gong, Z. Lin

Institute of Systems Biomedicine, Department of Pathology, Department of Biophysics School of Basic Medical Sciences, Peking University Health Science Center

Beijing 100191, P. R. China

E-mail: zhiqiang_lin@bjmu.edu.cn

Q. Xie

Division of Nephrology, Peking University Third Hospital

Beijing, 100191, P.R. China

J. Sun

Jiangsu Key Laboratory for Biomaterials and Devices, School of Biological Science and Medical Engineering, Southeast University

Nanjing, 210096, P. R. China

H. Tang

Department of Computer Science, Peking University

Beijing, 100191, P.R. China

Z. Xu

State Key Laboratory of Natural and Biomimetic Drugs, School of Pharmaceutical Sciences, Peking University

Beijing, 100191, P. R. China

W. Wang

Department of Rehabilitation Radiology, Beijing Rehabilitation Hospital, Capital Medical University

Beijing 100144, P. R. China

H. Xiao

Beijing National Laboratory for Molecular Science State Key, Laboratory of Polymer Physics and Chemistry, Institute of Chemistry, Chinese Academy of Science

Beijing 100190, P. R. China

E-mail: [hhxiao@iccas.ac.cn](mailto:hhxiao@iccas.ac.cn)

H. Han

Department of Radiology, Peking University Third Hospital

Institute of Medical Technology, Peking University Health Science Center

Beijing 100190, P. R. China

E-mail: [hanhongbin@bjmu.edu.cn](mailto:hanhongbin@bjmu.edu.cn)

S. Zhang and K. Shang contributed equally to this work.

*Corresponding Author: H. Han ([hanhongbin@bjmu.edu.cn](mailto:hanhongbin@bjmu.edu.cn)), Z. Lin (zhiqiang_lin@bjmu.edu.cn), H. Xiao ([hhxiao@iccas.ac.cn](mailto:hhxiao@iccas.ac.cn))

**Additional Experimental Section**

**1. Materials**

Poly (ethylene glycol) methyl ether (average Mw 5000, mPEG113), and 2-bromoisobutyryl bromide were purchased from Adamas-beta (CHN). 1-(2-Hydroxyethyl) piperidine (C6-OH), methacryloyl chloride, benzyl methacrylate (Bn-MA), 2,2'-Azobis(2-methylpropionitrile) (AIBN) were purchased from Tokyo Chemical Industry (TCI, JPN). Trimethylamine, n-hexane, Manganese chloride tetrahydrate, phosphoric acid, and 2-aminoethyl methacrylate (AMA) were purchased from Aladdin (USA). 1,1'-dioctadecyl-3,3,3',3'-tetramethylindocarbocyanine perchlorate (DiI) was purchased from MedChemExpress (USA). Cyanine5 N-hydroxysuccinimide ester was purchased from Shanghai Acmec Biochemical Technology Co., Ltd. Hydrogenated soy phosphatidylcholine (HSPC) was purchased from Nippon Fine Chemical. Phosphatidylserine was purchased from Shanghai Yuanye Bio-Technology Co., Ltd. Cell-Counting-Kit-8 (CCK-8) was purchased from New Cell &Molecular Biochemical Technology Co., Ltd. Solvents were purchased from Sinopharm Chemical Reagent Co., Ltd and used as received unless otherwise specified.

TNF-α (E-EL-M3063), IFN-β (E-EL-M0033), IFN-γ (E-EL-M0048), and IL-10 (E-EL-M0046) Elisa kit were purchased from Elabscience Biotechnology Co., Ltd. Antibodies used in this study include Tubulin (proteintech, 11224-1-AP) STING (Solarbio, K114101P), P-STING (Cell Signaling Technology, 50907), TBK1 (Cell Signaling Technology, 3504), P-TBK1 (Cell Signaling Technology, 5483T), IRF3 (Cell Signaling Technology, 29047), P-IRF3 (Cell Signaling Technology, 29047), IRF3 (Cell Signaling Technology, 4302). HRP-conjugated anti-rabbit secondary antibody (Proteintech, SA00001-2, 1:5000), Alexa Fluor 555-conjugated Goat anti-rabbit IgG (Abcam, ab150078). Fluorescent antibodies used in this study include Brilliant Violet 421-anti-mouse CD80 (Biolegend, 104725), PerCP-anti-mouse CD86 (Biolegend, 105025), PE/Cyanine7-anti-mouse CD11C (Biolegend, 117317), Brilliant Violet 510-anti-mouse F4/80 (Biolegend, 123135), Brilliant Violet 605-anti-mouse CD4 (Biolegend, 100548), Brilliant Violet 650-anti-mouse CD8 (Biolegend 100741,) PE-anti-mouse CD3 Antibody (Abclonal, A26897).

**2. Cell lines**

GL261 mouse glioma cell lines and BV2 mouse microglial cells were purchased from MeisenCTCC (CHN). The cells were cultured in DMEM/F12 supplemented with 10% fetal bovine serum (FBS) and 1% (v/v) penicillin-streptomycin (100 μg/ml) at 37°C in a 5% CO_2_ atmosphere.

**3. Synthesis of organic-inorganic copolymer**

*3.1. Synthesis of manganese phosphate ionic oligomers (MnP)*

MnP was prepared according to previous reports.^[1]^ In brief, 0.37 g of MnCl_2_·4H_2_O was dissolved in 500 ml of ethanol, and TEA (5.56 ml) was added to the above solution under magnetic stirring for 30 min at 25°C. The H_3_PO_4_ alcohol solution (0.6 ml of H_3_PO_4_ dissolved in 10 ml of ethanol) was then added dropwise under magnetic stirring for 12 h at 25℃. The MnP oligomers were concentrated by centrifugation (8000 rpm, 6 min) and washed with ethanol several times to remove the impurities. The MnP ionic oligomers were redispersed in ethanol to form a homogeneous slurry for future use.

*3.2. Synthesis of PC6AB*

C6-OH and trimethylamine were dissolved in 13 ml of THF, and methacryloyl chloride was added dropwise into the flask at 0℃. The reaction mixture was refluxed at 75℃ for 6 h. After the reaction, the mixture was filtered to remove the precipitate, and THF was evaporated under reduced pressure, yielding a yellow liquid (methacrylic acid 2-piperidinoethyl ester, C6-MA).^[2]^

PC6AB copolymers were synthesized by atom transfer radical polymerization (ATRP). mPEG_113_-Br was synthesized and purified according to the literature.^[3]^ mPEG_113_-Br (100.0 mg, 0.02 mmol), C6-MA (331.2 mg, 1.60 mmol), Bn-MA (70.5 mg, 0.40 mmol), AMA-MA (33.1 mg, 0.20 mmol), and AIBN (65.7 mg, 0.04 mmol) were dissolved in 1,4-dioxane (6 ml). After the removal of oxygen by injecting argon into the system, the mixture was stirred at 75℃ for 20 h. The reaction product was precipitated in cold hexane to remove impurities, and the precipitate was collected by filtration and dried under vacuum overnight to obtain a yellow solid.

*3.3. Synthesis of Cy5- and MnP-labeled PC6AB*

For the synthesis of PC6AB-MnP-Cy5, PC6AB, Cy5-NHS, and MnP were dissolved in a 2 ml mixture of ethanol and dichloromethane (1/1, v/v). The reaction was performed at room temperature for 24 h in the dark and was distilled under reduced pressure to remove the solvent and obtain the product.

**4. Preparation of nanoparticles**

PC6AB (5 mg) was dissolved in 1 ml of THF, and the resulting solution was slowly added dropwise to 9 ml of distilled water under ultrasonic conditions to form encapsulated nanoparticles. The nanoparticles were then filtered through an ultrafiltration tube with a molecular weight of 10 kDa and washed 5 times with distilled water to remove organic solvents and impurities. The final nanoparticle suspension was adjusted to a volume of 1 ml with distilled water.

**5. Characterization of nanomaterials**

Bruker AmaZon ion trap mass spectrometer (Bruker, USA) which was equipped with an electrospray ionization (ESI) source was used to measure the molecular weight of MnP. X-ray photoelectron spectroscopy (XPS, ThermorFisher, USA) analyzed the molecular structure information in MnP. Inductively Coupled Plasma Optical Emission Spectrometry (ICP-OES, ThermorFisher, USA) was used to quantify the mass fraction of manganese and phosphorus in MnP. Deuterated chloroform solutions of polymers were sealed in NMR tubes and immediately measured 400 MHz NMR spectrometer (Zhongke-Niujin, CHN) at room temperature for the ^1^H spectra in order to analyze the structure of the polymer. The X-ray powder diffraction (Rigaku, JPN), attenuated total reflectance-fourier transform infrared (ATR-FTIR, Nicolet, USA), and differential scanning calorimetry (DSC, Netzsch, GER) spectra of MnP, NP, and NP-MnP nanoparticles were recorded. Dynamic light scattering (DLS) measured their size and Zeta potential. Scanning electron microscope (SEM, ThermorFisher, USA) and transmission electron microscope (TEM, JEOL, JPN) were used to observe the topography and elemental analysis of NP at pH 7.4 and 6.8.

**6. pH-dependent properties and membranolytic selectivity of nanoparticles**

*6.1. pH-responsive property of the nanoparticles in vitro conditions*

Different concentrations of MnP (0.13–1.0 mM) solutions were analyzed using a 3.0 T MRI scanner (GE, USA). To examine the MRI related to acid-triggered nanoparticle dissociation, PBS of different pH (6.4, 6.6, 6.8, 7.0, 7.2, 7.4) was used to dilute the nanoparticles (with Mn concentration set at 0.25 mM) for MRI. To investigate fluorescence imaging of nanoparticles in the acidic environment, the nanoparticle was diluted to 0.1 mg/ml with PBS at different pH (6.4, 6.6, 6.8, 7.0, 7.2, 7.4). Fluorescence images were recorded using a near-infrared imaging system (PerkinElmer, USA) at an excitation wavelength of 630 nm.

*6.2. In vitro cytotoxicity*

Cytotoxicity was assessed using the CCK-8 assay. GL261 and BV2 cells were seeded into 96-well plates at a density of 8×10^3^ cells per well. After incubation for 24 h, the culture medium was replaced with different concentrations of NP-MnP and incubated for different durations under predetermined pH conditions (pH 6.8 or 7.4). The medium was replaced with the medium solution of CCK8 and incubated for another 2 h. Absorbance of the solution in each well was measured at 450 nm using the microplate reader (Molecular Devices, USA).

*6.3. SEM observation of NP-MnP treated cells*

GL261 and BV2 cells were seeded onto 12-well plates with circular glass slides at a density of 10^5^ cells per well. NP-MnP was applied at a concentration of 50 μg/ml for 1h at pH 6.8 or pH 7.4. The cells were fixed with glutaraldehyde for 30 min. The samples were dried using the critical point drying method and then observed using SEM.

*6.4. TEM observation of NP-MnP treated cells*

GL261 and BV2 cells were seeded onto 100 mm dishes at a density of 5×10^5^ cells per dish. After incubation for 24 h, the media was replaced by DMEM/F12 containing NP-MnP (50 μg/ml) at predetermined pH conditions (pH 7.4 or 6.8). The cells were collected at specific time points, fixed with 2.5% glutaraldehyde for more than 4 h, and then observed using TEM.

*6.5. Determination of the dynamic interaction between NP-MnP-Cy5 and cells*

GL261 cells were seeded onto glass-bottomed cell culture dishes at a density of 2×10^5^ cells per dish. After incubation for 24 h, the cell membranes were firstly stained with DiI, and then DMEM/F12 containing NP-MnP-Cy5 (50 μg/ml) was added instead of the medium at pH 6.8. The cells were monitored for 1 h using an oil-immersed ×40 objective lens on a CLSM (Leica, Germany).

*6.6. Construction of two kinds of liposomes mimicking tumor cells and normal cells*

Phosphatidylserine (PS), cholesterol, DSPE-PEG, and tetramethylrhodamine (TMR) were dissolved in chloroform, mixed thoroughly and the organic solvents were removed by distillation under reduced pressure. Liposomes mimicking normal cells were obtained by adding 1 ml of phosphate buffer under sonication conditions. Liposomes mimicking tumor cells were prepared by dissolving PS, cholesterol, DSPE-PEG, HSPC, and TMR in chloroform using the same method as above. A volume of 20 μl from each liposome type was taken, and the fluorescent spots were observed using CLSM. The number of fluorescent spots was quantified after co-incubation with NP-MnP under predetermined pH conditions (pH 7.4 or 6.8).

**7. In vitro analysis of immune response**

*7.1. Western blot analysis of STING signaling pathways assay*

GL261 cells were incubated in 6-well plates at a density of 10^6^ cells per well after overnight incubation. The cells were then treated with 1) PBS, 2) NP, 3) MnP, 4) NP-MnP, 5) ADU, 6) NP-MnP-ADU for 24 h. The final concentrations of the nanoassemblies were maintained at an equivalent concentration of 50 μg/ml for NP, 21 μg/ml for Mn, and 2 μg/ml for ADU. Supernatants of GL261 cells were collected and incubated with BV2 cells for another 24 h, for which the BV2 cells were seeded in 6-well plates at a density of 10^6^ and pre-incubated for 24 h. The proteins from the BV2 cells were extracted, and protein concentrations were determined using a BCA protein assay kit. Equal amounts of protein were separated by SDS polyacrylamide gel electrophoresis and transferred to a polyvinylidene fluoride (PVDF) membrane. The membrane was blocked with Phosphate Buffered Saline with Tween 20 (PBST) solution containing 5% bovine serum albumin (BSA) and subsequently incubated overnight with primary antibodies, including STING, P-STING, TBK1, P-TBK1, IRF3, and P-IRF3. After incubation with a goat anti-rabbit IgG secondary antibody for 1 h, protein bands were detected using ECL substrate and chemiluminescence.

*7.2. Immunofluorescence characterization using CLSM*

GL261 cells were incubated in 12-well plates at a density of 10^6^ cells per well overnight and then 1) PBS, 2) NP, 3) MnP, 4) NP-MnP, 5) ADU, 6) NP-MnP-ADU were added and incubated for an additional 24 h. The supernatants of the GL261 cells were collected and transferred to BV2 cells, which had been seeded onto cover slips at a density of 10^5^ cells per slide and incubated for an additional 24 h. Then, the cells were fixed in the 4% paraformaldehyde solution, blocked with 1% BSA and incubated with 0.1% Triton. Afterward, the cells were incubated with P-STING, P-TBK1, and P-IRF3 antibodies, diluted in cell culture media, at 4℃ for 12 h. The cells were further incubated with the secondary Alexa Fluor-555-conjugated antibodies for 2 h. The cell nuclei ware stained with DAPI, and images were taken with a CLSM.

*7.3. ELISA Assay on the biochemical alterations of nanoagonist-stimulated immune cells*

GL261 cells (10^5^) and BV2 cells (10^5^) were co-cultured in a 12-well plate overnight. The culture medium was then replaced with fresh medium containing 1) PBS, 2) NP, 3) MnP, 4) NP-MnP, 5) ADU, 6) NP-MnP-ADU and cells were further cultured for 24 h. The supernatant was collected, centrifuged at 12000 rpm for 5 min, and measured the levels of TNF-α, TNF-γ, and IFN-β cytokines in the sample using an ELISA kit.

*7.4. Flow cytometry analysis on the maturation and activation status of immune cells in vitro*

GL261 cells were seeded in 12-well plates at a density of 3×10^5^ cells per well and incubated overnight. The GL261 cells were treated with the following conditions for 24 h. 1) PBS, 2) NP, 3) MnP, 4) NP-MnP, 5) ADU, 6) NP-MnP-ADU. The culture supernatant was subsequently collected and incubated with BV2 cells for 24 h. Afterward, the BV2 cells were collected and stained with fluorescent antibodies as follows: Brilliant Violet 421-anti-mouse CD80, PerCP-anti-mouse CD86, PE/Cyanine7 -anti-mouse CD11C, Brilliant Violet 605-anti-mouse CD4, Brilliant Violet 650-anti-mouse CD8, PE-anti-mouse CD3. The maturation of immune cells was assessed by flow cytometry.

*7.5. RNA Sequencing Analysis*

GL261 cells (5×10^5^) and BV2 cells (5×10^5^) were co-cultured in 100 mm dishes overnight. The cells were treated with PBS, NP, MnP, NP-MnP, ADU, and NP-MnP-ADU for 24 h, respectively. The cell samples from different treatments were collected to purify RNA. Differentially expressed genes were identified with fold changes ≥ 2 and q-values ≤ 0.05. GO and KEGG enrichment analysis was conducted with the R phyper, and significant enrichments were defined as q-values ≤ 0.05.

**8. In vivo antitumor effect of different nanoparticles**

*8.1. Construction of animal models and therapeutic evaluation*

To establish a glioma model, C57BL/6 mice were anaesthetized with tribromoethanol and then restrained using a stereotaxic frame for aseptic survival surgery and craniotomy. A hole was drilled in the left caudate nucleus (2.0 mm lateral, 1.0 mm anterior to the bregma, and 3.0 mm deep) and 2×10^5^ GL261 cells were injected into the caudate nucleus at 0.5 µl/min. Tumors grew for 5 d before receiving different treatments. All animal experiments were conducted in accordance with ethical guidelines and approved by the Ethics Committee.

Mice were divided into 6 groups randomly and administered the following treatments: 1) PBS, 2) NP, 3) MnP, 4) NP-MnP, 5) ADU, 6) NP-MnP-ADU (n = 4, NP: 15 μg/mouse, MnP: 9 μg/mouse, and ADU: 10 μg/mouse). The volume of different nanoparticles was consistent at 3 μl, and the administration was performed via CED at a rate of 0.5 µl/min every 5 days. Body weight and tumor volume were recorded every 5 days. Tumor growth was monitored by 3T MRI scanner. T2-weighted imaging (T2WI) MRI scans were performed on the mice's brains. The tumor area appeared as a mass of abnormal high signal on T2WI images. Tumor volume was determined using the imaging data. Tumor contours were outlined on each slice using software (RadiAnt DICOM Viewer) to obtain the tumor area. The areas of the tumor on the n-th image were denoted as S1, S2, S3, ..., Sn, and the slice thickness was represented by ST. Tumor volume (V) was then calculated using the formula: V = (S1 + S2 + S3 + ... + Sn) × ST.

*8.2. In vivo biocompatibility evaluation*

At the end of immunotherapy, serum samples were collected by centrifugation whole blood (4000 rpm, 10 min) to measure the concentrations of AST, ALT, urea, and UA. All mice were sacrificed, and their major organs were collected, fixed with 4% paraformaldehyde, and embedded in paraffin. Tissue sections were stained with H&E and observed under an optical microscope.

**9. In vivo imaging for biodistribution analysis**

*9.1. In vivo MRI*

For in vivo MRI, the GL261 tumor-bearing mice were administered NP-MnP-Cy5 (3 μl) via CED. T1-weighted images were acquired at 0, 1, 2, and 3 h post-injection using a clinic 3T MRI scanner (GE750, TR = 300 ms, slice thickness = 2.0 mm). The MRI signal intensity before injection was used as the baseline control (n = 3).

*9.2. In vivo fluorescence imaging and biodistribution*

GL261 tumor-bearing mice were administered NP-MnP-Cy5 (3 μl) via CED for in vivo fluorescence imaging. Fluorescence signals were recorded using the IVIS imaging system at 0, 1, 2, and 3 h after the injection, with the pre-injection signal serving as the control. To evaluate the distribution of NP-MnP-Cy5, mice were sacrificed 48 h post-injection. Main organs (liver, heart, lung, spleen, tumor, and kidneys) were harvested for imaging and semi-quantitative biodistribution analysis (n = 3).

**10. In vivo immune response analysis**

*10.1. Analysis of the tumor immune microenvironment*

Fresh spleens and tumors were collected to prepare them into single-cell suspensions, which were then incubated with the relevant antibodies for 30 min at 4°C. To analyze the immune response of T lymphocytes in the spleen and tumor tissues, the cells were stained with anti-mouse CD3, anti-mouse CD4, and anti-mouse CD8. To analyze the maturation of immune cells in the tumors and spleens, cells were stained with anti-mouse CD11C, anti-mouse CD80, and anti-mouse CD86. To assess the proportion of infiltrating macrophages in the tumor tissue, cells were stained with anti-mouse F4/80, anti-mouse CD80 antibody. Flow cytometry data were collected using CytExpert software and FlowJo software was used to process the data.

*10.2. ELISA assay of the serum*

Peripheral blood of mice treated with different drugs was collected and allowed to stand at room temperature for 2 h. The blood was then centrifuged at 4000 rpm for 10 min, and the supernatant was collected. The levels of cytokines IFN-β, IFN-γ, IL-10, and TNF-α in the above peripheral blood were measured using ELISA kits to evaluate the in vivo immune response.

*10.3. Immunofluorescence analysis of tumor samples*

After treatments, mice were euthanized, and their brains were extracted for sectioning. The brain slices were incubated overnight with primary antibodies P-STING, P-TBK1, and P-IRF3, respectively, followed by staining with fluorescent secondary antibodies and DAPI. The slices were analyzed using a digital slide scanner (3DHISTECH, HU) to assess the in vivo activation of the cGAS-STING*.*


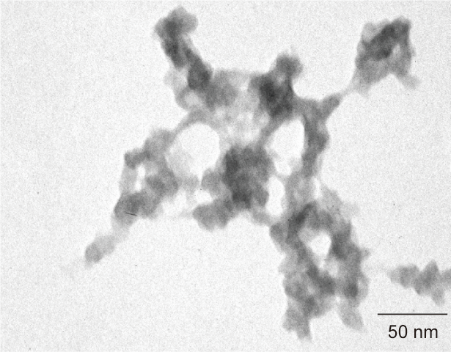


**Figure S1.** TEM image of the MnP for illustrating the morphological details.


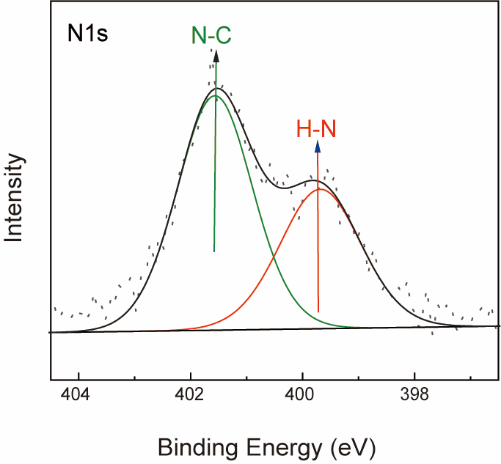


**Figure S2.** XPS spectra of N1s of MnP.


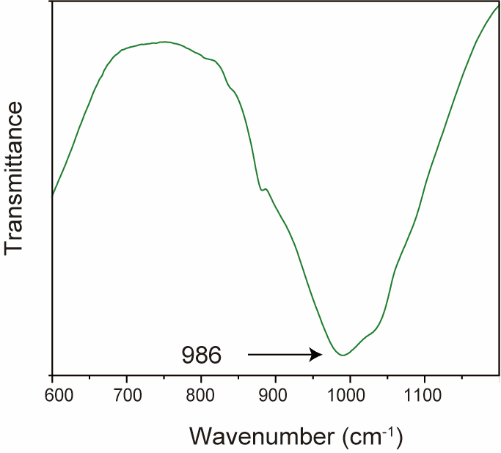


**Figure S3.** ATR-FTIR spectrum of MnP.


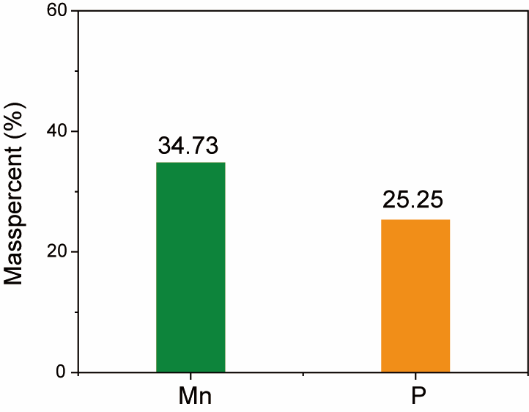


**Figure S4.** Atomic mass fraction of Mn and P of the MnP ionic chains, as determined by ICP-OES.


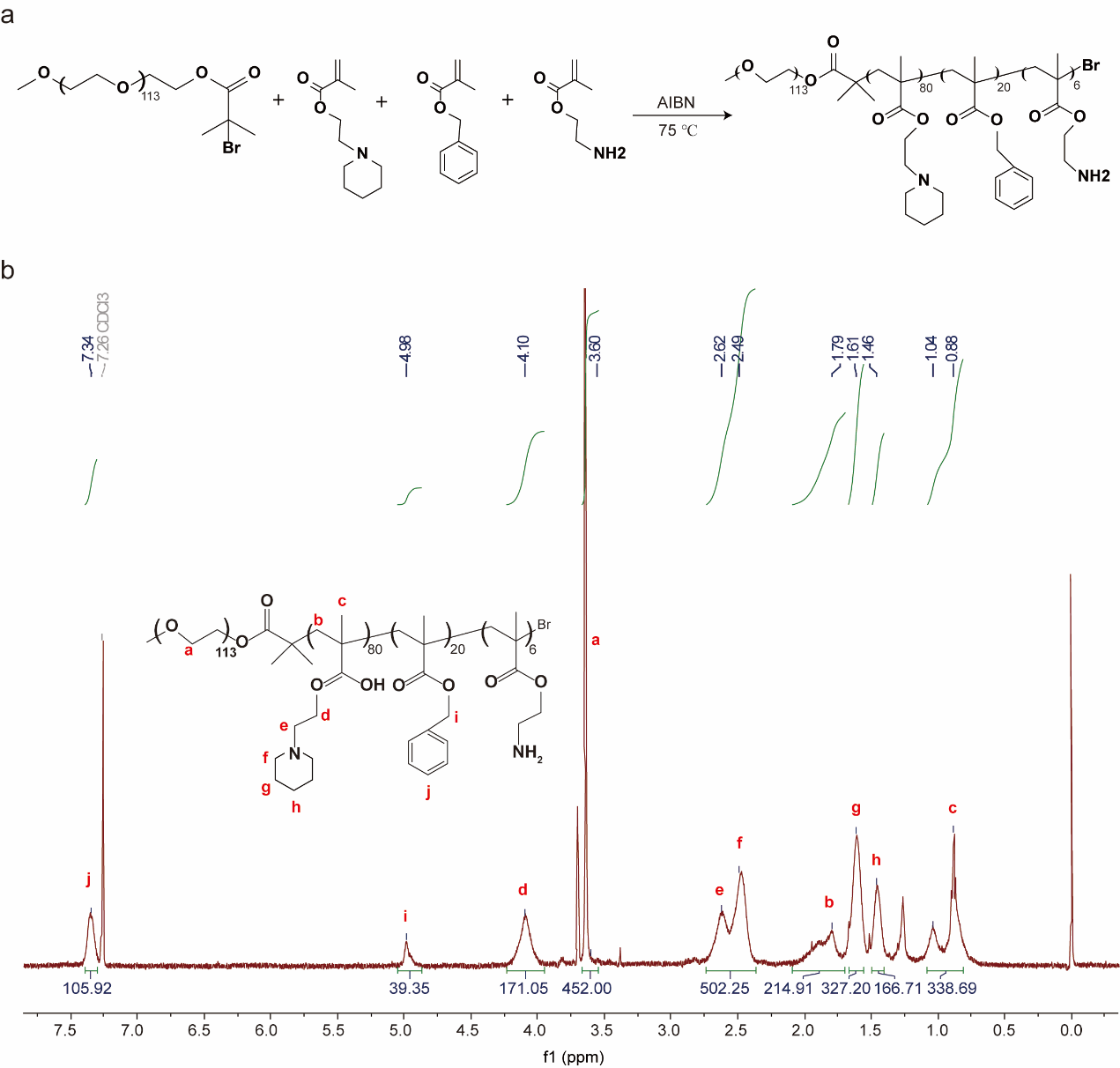


**Figure S5.** a) The synthetic process of the tri-block copolymers by ATRP polymerization. b) The ^1^H NMR spectra of PC6AB.


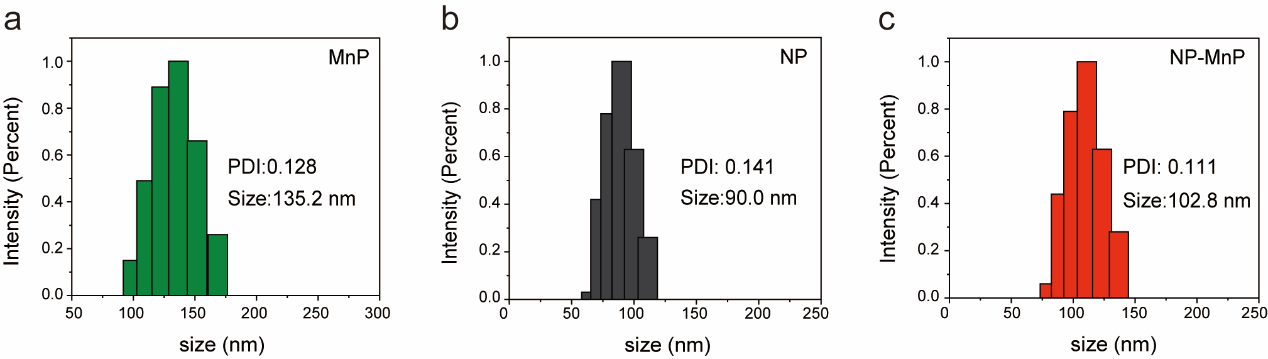


**Figure S6.** a-c) DLS analysis of the size distribution of MnP (a), NP (b), and NP-MnP (c).


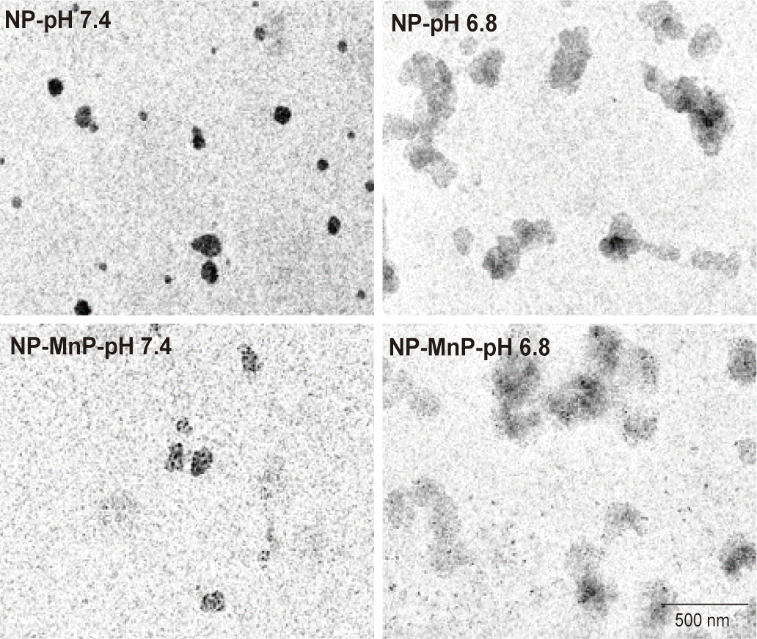


**Figure S7.** Transmission electron micrographs of NP-MnP and NP at pH 7.4 and 6.8 (polymer concentration, 10 μg/ml).


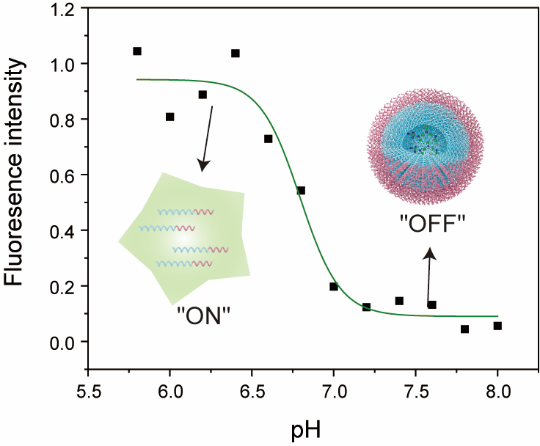


**Figure S8.** Normalized fluorescence intensity as a function of pH for NP-MnP.


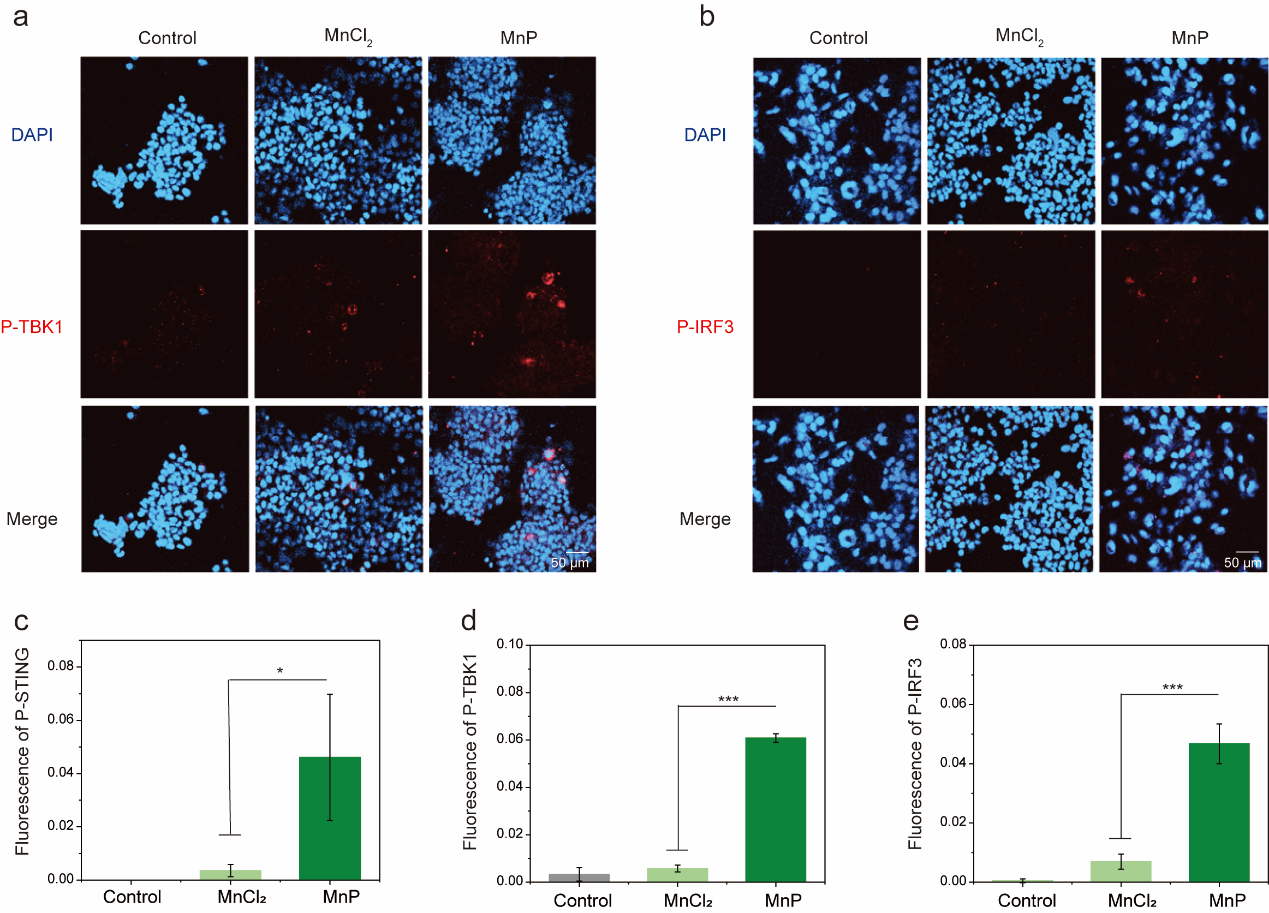


**Figure S9.** a-b) Immunofluorescence CLSM images of P-TBK1 (a) or P-IRF3 (b) upon treatments. c-e) Quantitative fluorescence analysis of P-STING (c), P-TBK1 (d), and P-IRF3 (e) expression levels. All data were expressed as mean ± SD (n = 3). Statistical analysis was carried out via Student's t-test. The significance levels were indicated as follows: * P < 0.05, *** P < 0.001.


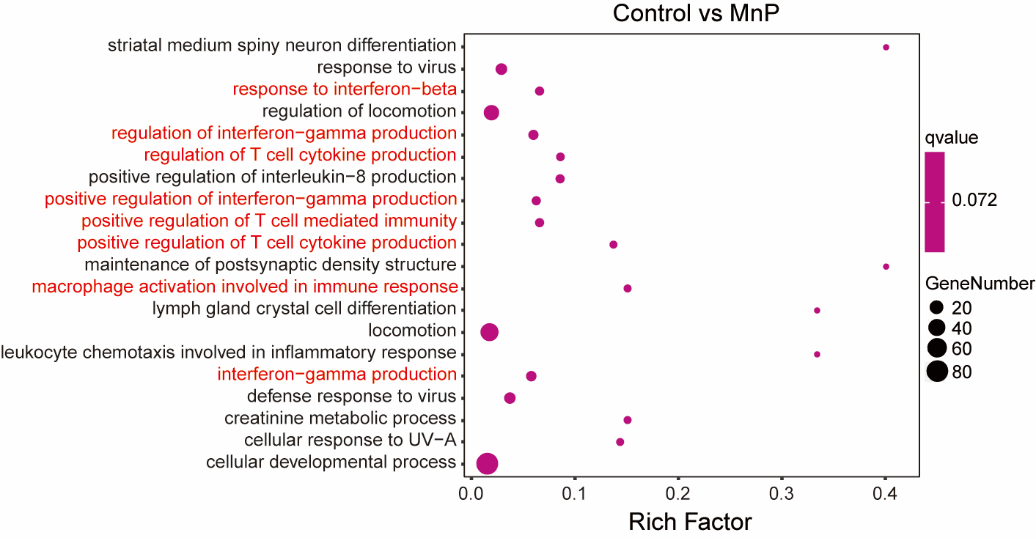


**Figure S10.** GO analysis of differentially expressed genes between cells treated with PBS and MnP.


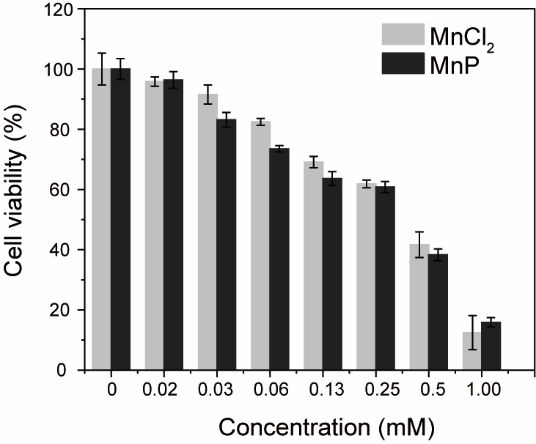


**Figure S11.** The concentration-dependent cytotoxicity of MnCl_2_ or MnP after 24 h of incubation. All data were expressed as mean ± SD (n = 3). Statistical analysis was carried out via two-way ANOVA method. There is no significant difference in toxicity between MnP and MnCl_2_.


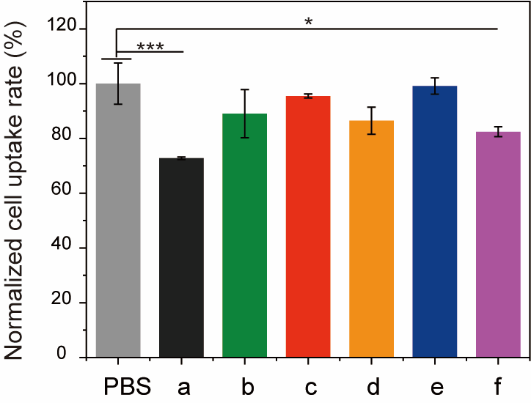


**Figure S12.** Cell uptake of NP-MnP-Cy5 in BV2 cells after pre-treatment with glucose (a), methyl-β-cyclodextrin (b), colchicine (c), wortmannin (d), chlorpromazine hydrochloride (e), and nystatin (f). All data were expressed as mean ± SD (n = 3). Statistical analysis was carried out via one-way ANOVA method. The significance levels were indicated as follows: * P < 0.05, *** P < 0.001. The cell uptake inhibitors and their corresponding mechanisms included glucose (energy-dependent), methyl-β-cyclodextrin (caveolae-dependent), colchicine (microtubule-dependent), wortmannin (macropinocytosis), chlorpromazine hydrochloride (clathrin-dependent), and nystatin (caveolin-dependent).


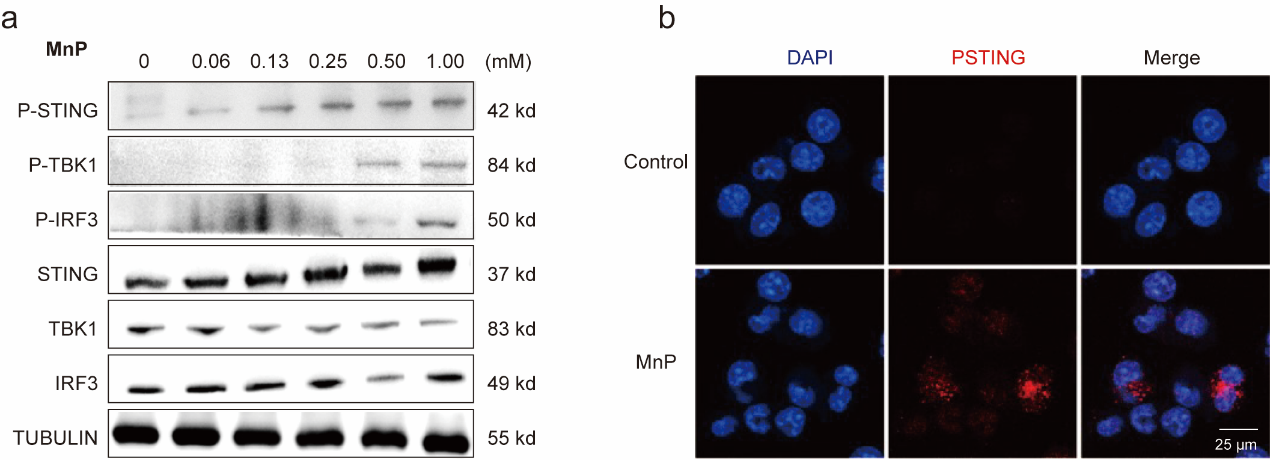


**Figure S13.** a) The expression levels of STING pathway associated proteins upon concentration dependent treatment with MnP determined by western blot analysis. b) Immunofluorescence CLSM images of P-STING in GL261 cells upon treatment.


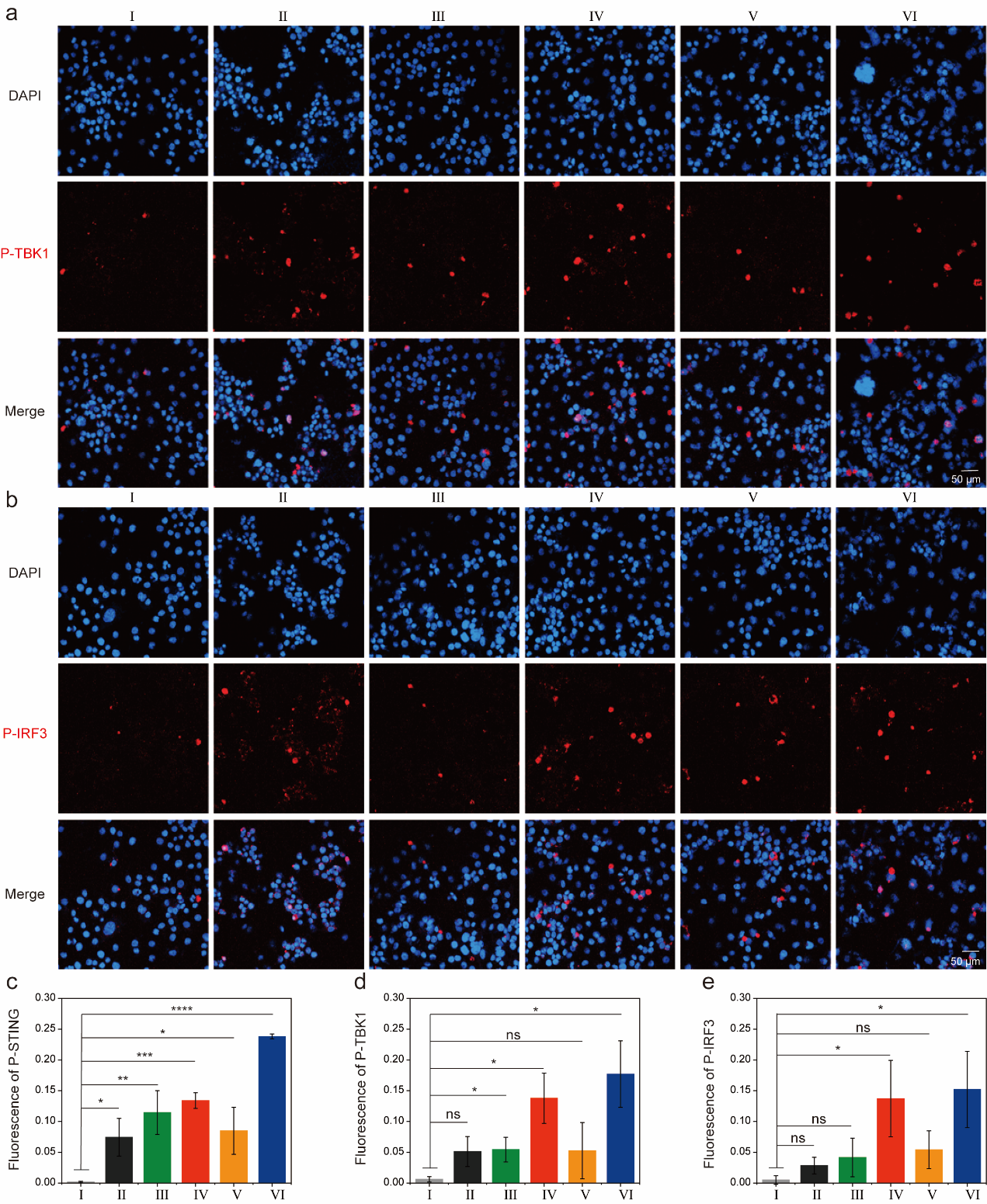


**Figure S14.** a-b) CLSM images of P-TBK1 (a) or P-IRF3 (b) immunofluorescence in BV2 cells after different treatments, including: (I) Control, (II) NP, (III) MnP, (IV) NP-MnP, (V) ADU, and (Ⅵ) NP-MnP-ADU. c-e) Quantitative fluorescence analysis of P-STING (c), P-TBK1 (d), and P-IRF3 (e) expression levels. All data were expressed as mean ± SD (n = 3). Statistical analysis was carried out via one-way ANOVA method. The significance levels were indicated as follows: ns (no significance), * P < 0.05, ** P < 0.01, *** P < 0.001, **** P < 0.0001.
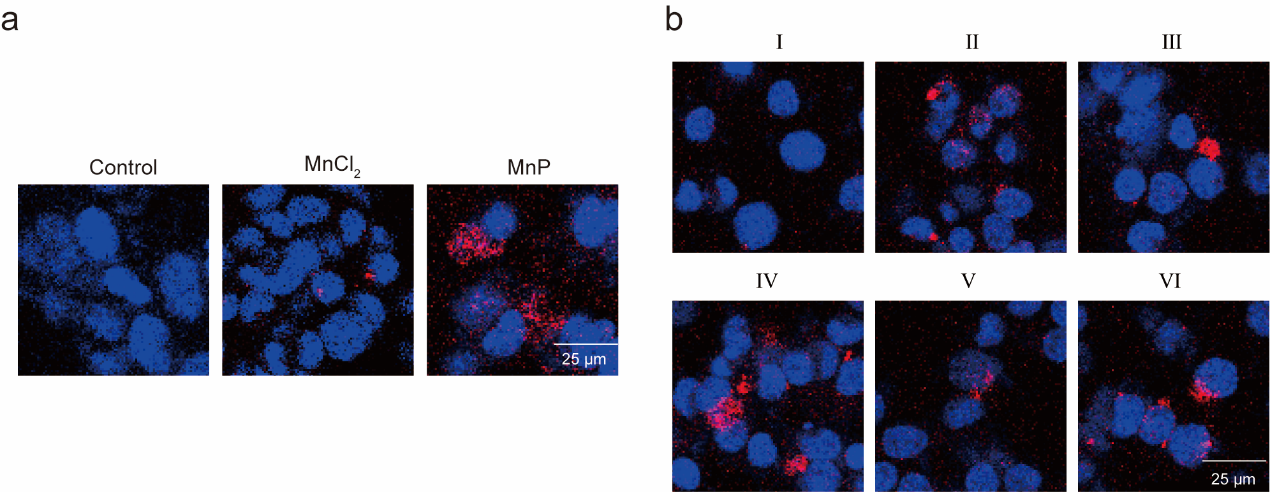


**Figure S15.** The partial zoom-in on the merge images in Figure S9 (b) and S14 (b). Different treatments including: (I) Control, (II) NP, (III) MnP, (IV) NP-MnP, (V) ADU, and (Ⅵ) NP-MnP-ADU. Red: P-IRF3, Blue: nuclei.


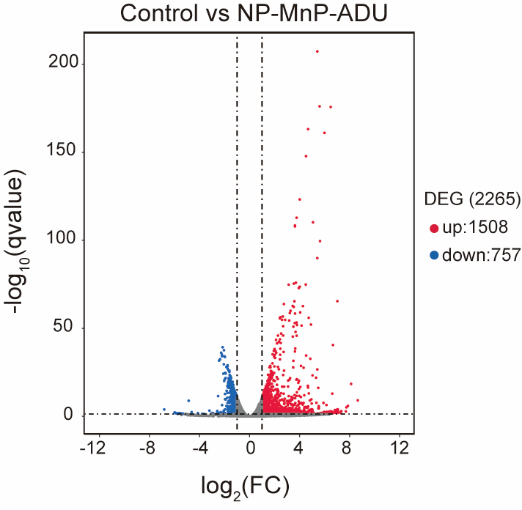


**Figure S16.** Volcano plots displayed the differentially expressed genes between cells treated with PBS and NP-MnP-ADU.


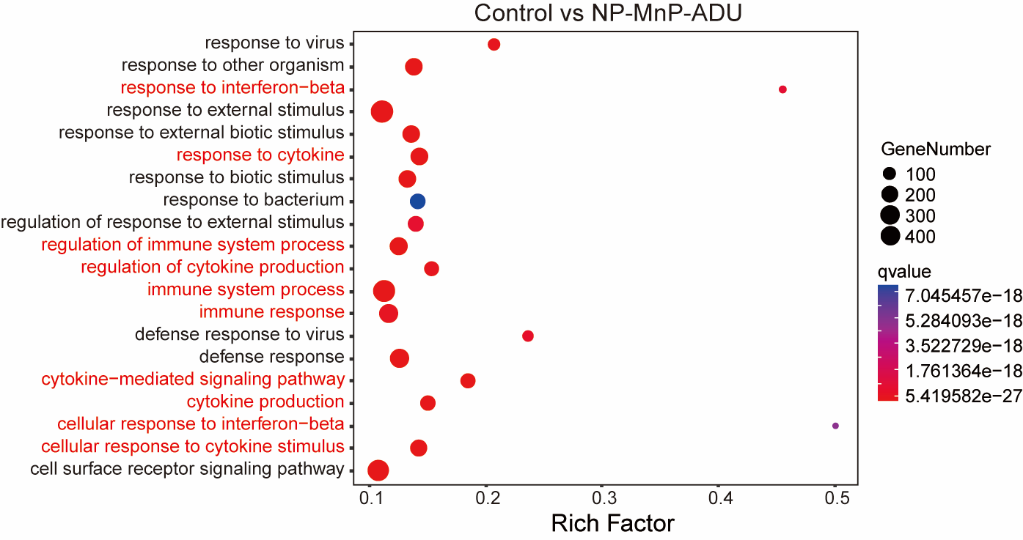


**Figure S17.** GO analysis of differentially expressed genes between cells treated with PBS and NP-MnP-ADU.


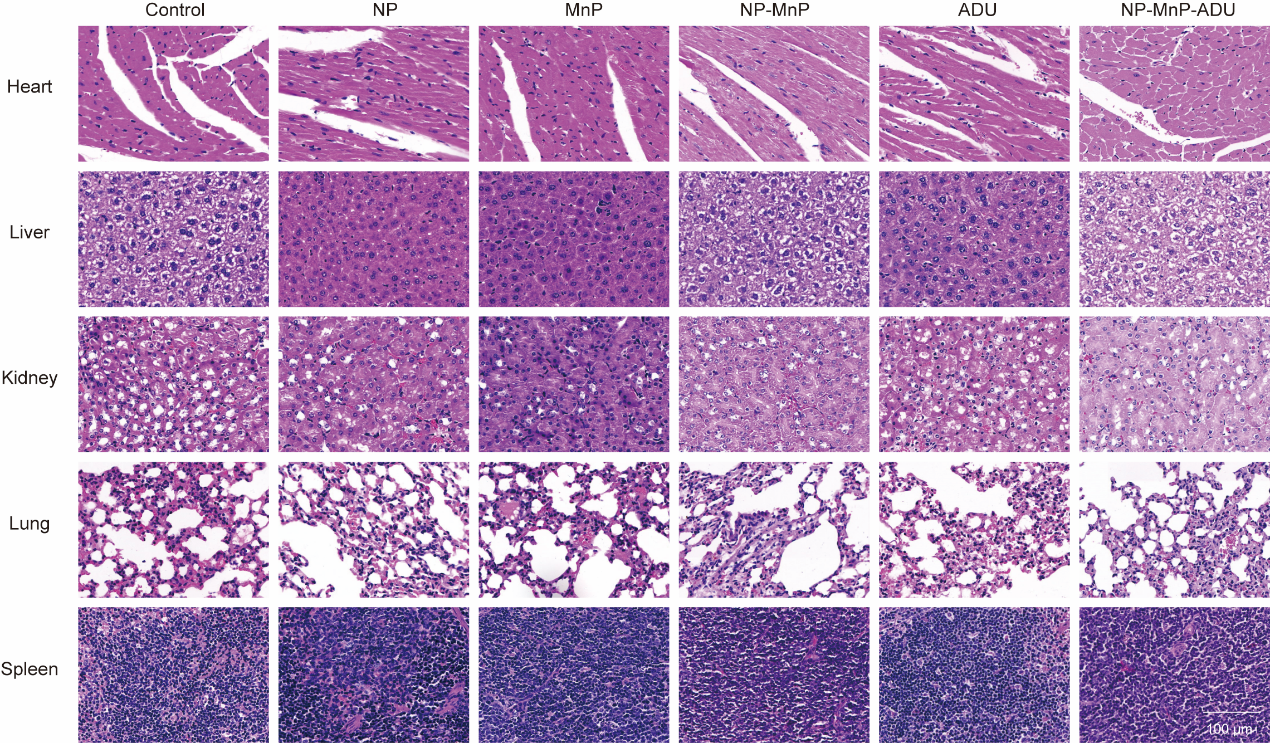


**Figure S18.** In vivo systemic toxicity after different treatments to GL261 tumor-bearing mice. H&E staining images of major organs dissected from treated mice.


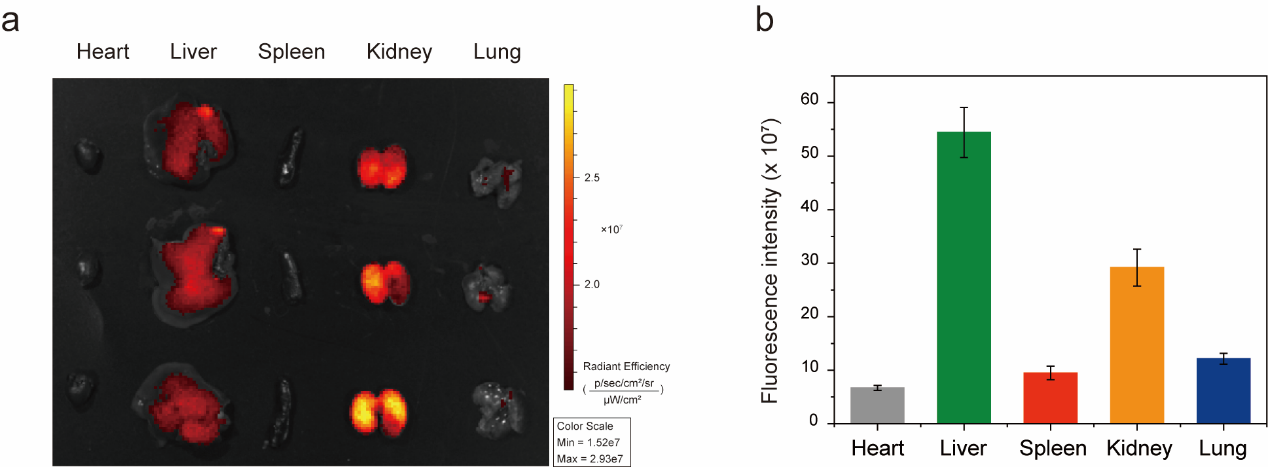


**Figure S19.** a) Ex vivo fluorescence images of liver, spleen, lung, heart, and kidney were obtained 48 h after the administration of NP-MnP-Cy5 via CED. b) Quantitative fluorescence intensity analysis of major organs after a single-dose administration of NP-MnP-Cy5 via CED. All data were expressed as mean ± SD (n = 3).


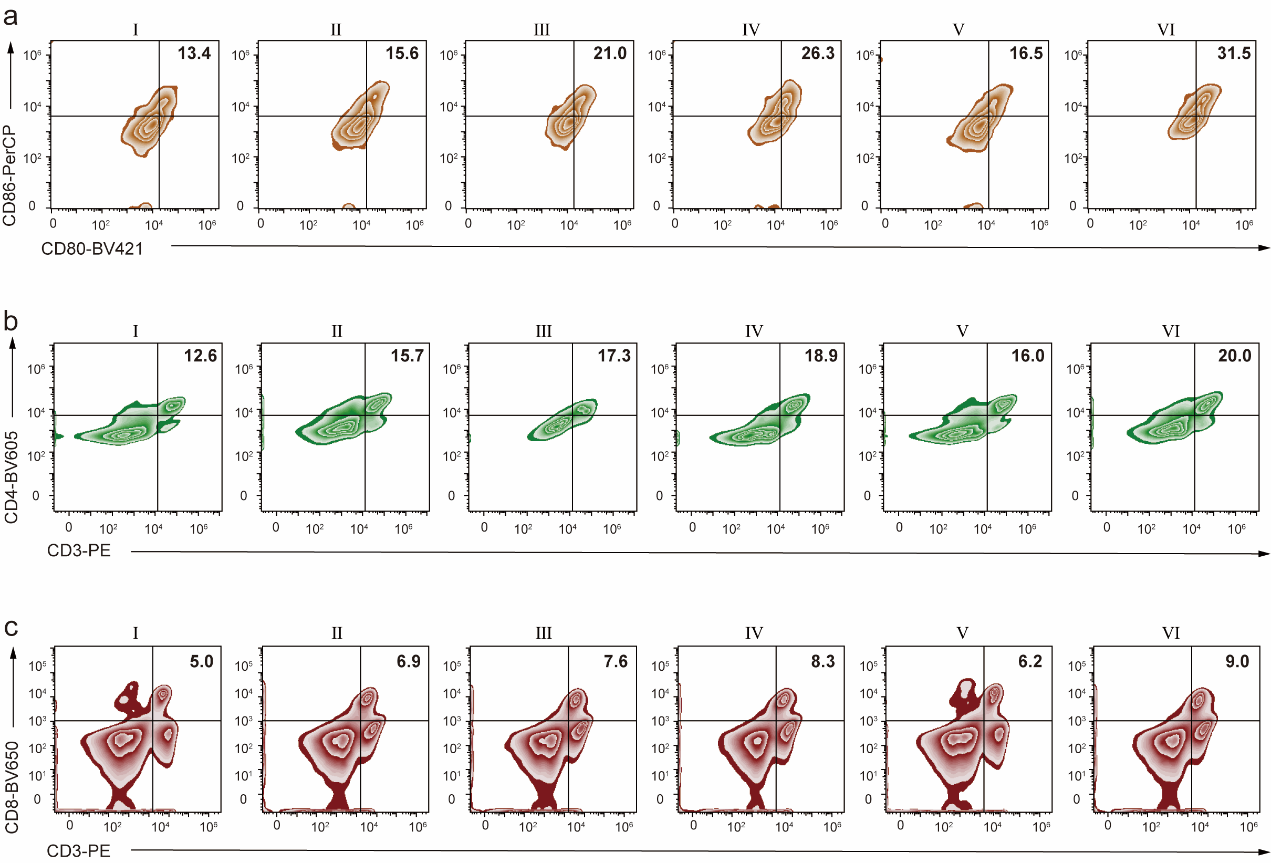


**Figure S20.** a-c) Representative flow cytometry images of the expression levels of CD80^+^/CD86^+^, CD3^+^/CD4^+^, and CD3^+^/CD8^+^ in GL261 tumor tissues after treatments with (I) Control, (II) NP, (III) MnP, (IV) NP-MnP, (V) ADU and (Ⅵ) NP-MnP-ADU in vivo. All flow cytometry experiments were repeated three times independently with similar results.


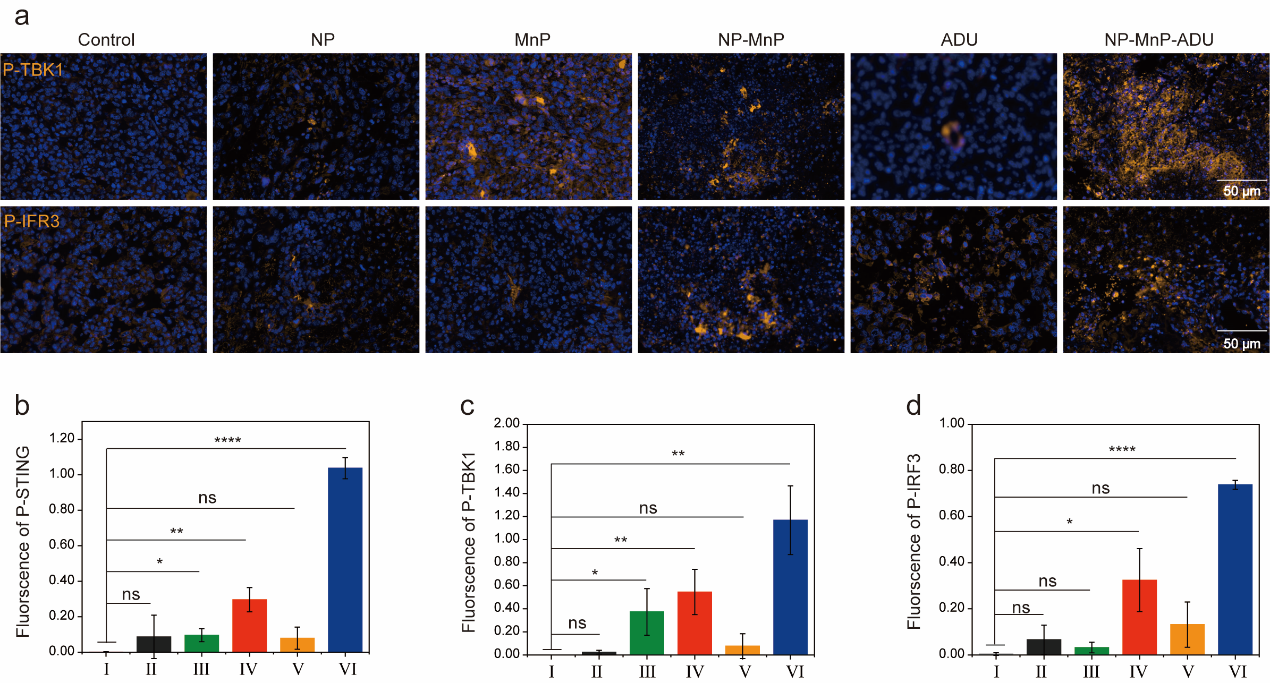


**Figure S21.** a) Immunofluorescence analysis of P-TBK1 and P-IRF3 expression levels in tumor tissues following different treatments, including: (I) control, (II) NP, (III) MnP, (IV) NP-MnP, (V) ADU, and (Ⅵ) NP-MnP-ADU. Yellow: P-TBK1/P-IRF3, Blue: nuclei. b-d) Quantitative fluorescence analysis of P-STING (b), P-TBK1 (c), and P-IRF3 (d). All data were expressed as mean ± SD (n = 3). Statistical analysis was carried out via one-way ANOVA method. The significance levels were indicated as follows: ns (no significance), * P < 0.05, ** P < 0.01, **** P < 0.0001.

**Supplementary References**

[1] Liu, Z., C. Shao., B. Jin., Z. Zhang., Y. Zhao., X. Xu., &R. Tang., Crosslinking ionic oligomers as conformable precursors to calcium carbonate, 2019, Nature, 574 (7778):394-398, http://doi.org/10.1038/s41586-019-1645-x

[2] Zhou, K., Y. Wang., X. Huang., K. Luby-Phelps., B. D. Sumer., &J. Gao., Tunable, ultrasensitive pH-responsive nanoparticles targeting specific endocytic organelles in living cells, 2011, Angew Chem Int Ed Engl, 50 (27):6109-14, http://doi.org/10.1002/anie.201100884

[3] Sun, Yizhe., Lidong. Gong., Yue. Yin., et al., A Gradient pH-Sensitive Polymer-Based Antiviral Strategy via Viroporin-Induced Membrane Acidification, 2022, Adv Mater, 34 (18):e2109580, http://doi.org/10.1002/adma.202109580
